# De novo* GRIN* variants in M3 helix associated with neurological disorders control channel gating of NMDA receptor

**DOI:** 10.1007/s00018-023-05069-z

**Published:** 2024-03-28

**Authors:** Yuchen Xu, Rui Song, Riley E. Perszyk, Wenjuan Chen, Sukhan Kim, Kristen L. Park, James P. Allen, Kelsey A. Nocilla, Jing Zhang, Wenshu XiangWei, Anel Tankovic, Ellington D. McDaniels, Rehan Sheikh, Ruth K. Mizu, Manish M. Karamchandani, Chun Hu, Hirofumi Kusumoto, Joseph Pecha, Gerarda Cappuccio, John Gaitanis, Jennifer Sullivan, Vandana Shashi, Slave Petrovski, Robin-Tobias Jauss, Hyun Kyung Lee, Xiuhua Bozarth, David R. Lynch, Ingo Helbig, Tyler Mark Pierson, Cornelius F. Boerkoel, Scott J. Myers, Johannes R. Lemke, Timothy A. Benke, Hongjie Yuan, Stephen F. Traynelis

**Affiliations:** 1 Department of Pharmacology and Chemical Biology, Emory University School of Medicine, Atlanta, GA 30322 USA; 2 Center for Functional Evaluation of Rare Variants (CFERV), Emory University School of Medicine, Atlanta, GA 30322 USA; 3 Departments of Pediatrics and Neurology, University of Colorado School of Medicine and Children’s Hospital Colorado, Aurora, CO USA; 4 Department of Translational Medicine, Section of Pediatrics, Federico II University, Via Pansini 5, 80131 Naples, Italy; 5 Hasbro Children’s Hospital, The Warren Alpert Medical School of Brown University, Providence, RI USA; 6 Department of Pediatrics, Division of Medical Genetics, Duke University, Durham, NC USA; 7 Ce Department of Medicine, University of Melbourne, Austin Health, Melbourne, VIC Australia; 8 Centre for Genomics Research, Discovery Sciences, AstraZeneca, BioPharmaceuticals R&D, Cambridge, UK; 9 Institute of Human Genetics, University of Leipzig Medical Center, Leipzig, Germany; 10 Provincial Medical Genetics Program, Department of Medical Genetics, University of British Columbia, Children’s and Women’s Health Centre of BC, Vancouver, B.C V6H 3N1 Canada; 11 Center for Integrative Brain Research, Seattle Children’s Research Institute, Seattle, WA USA; 12 Division of Pediatric Neurology, Department of Neurology, Seattle Children’s Hospital, University of Washington, Seattle, WA USA; 13 Departments of Pediatrics and Neurology, Perelman School of Medicine, Children’s Hospital of Philadelphia, University of Pennsylvania, Philadelphia, PA USA; 14 Division of Child Neurology, The Children’s Hospital of Philadelphia, Philadelphia, PA 19104 USA; 15 Department of Pediatrics and Neurology, Cedars-Sinai Medical Center, Los Angeles, CA USA; 16 Center for the Undiagnosed Patient, Cedars-Sinai Medical Center, Los Angeles, CA USA; 17 Board of Governors Regenerative Medicine Institute, Cedars-Sinai Medical Center, Los Angeles, CA USA; 18 Center for Rare Diseases, University of Leipzig Medical Center, Leipzig, Germany; 19 Emory Neurodegenerative Disease Center, Emory University School of Medicine, Atlanta, GA 30322 USA; 20 Present Address: Department of Neurology, The First Hospital of Wenzhou Medical University, Wenzhou, Zhejiang 325000 China; 21 Present Address: Department of Neurology, Qilu Hospital of Shandong University, Jinan, Shandong 250012 China; 22 Present Address: Department of Psychiatry, Sir Run Run Shaw Hospital, Zhejiang University School of Medicine, Hangzhou, China; 23 Present Address: Department of Pediatrics and Pediatric Epilepsy Center, Peking University First Hospital, Beijing, China; 24 Present Address: Department of Pediatrics-Neurology, Baylor College of Medicine, Houston, TX USA; 25 Present Address: Jan and Dan Duncan Neurological Research Institute, Texas Children’s Hospital, Houston, TX USA

**Keywords:** Glutamate receptor, NMDA receptor, TMD, Channelopathy, Functional genomics

## Abstract

N-methyl-d-aspartate receptors (NMDARs) are members of the glutamate receptor family and participate in excitatory postsynaptic transmission throughout the central nervous system. Genetic variants in *GRIN* genes encoding NMDAR subunits are associated with a spectrum of neurological disorders. The M3 transmembrane helices of the NMDAR couple directly to the agonist-binding domains and form a helical bundle crossing in the closed receptors that occludes the pore. The M3 functions as a transduction element whose conformational change couples ligand binding to opening of an ion conducting pore. In this study, we report the functional consequences of 48 de novo missense variants in *GRIN1*, *GRIN2A*, and *GRIN2B* that alter residues in the M3 transmembrane helix. These de novo variants were identified in children with neurological and neuropsychiatric disorders including epilepsy, developmental delay, intellectual disability, hypotonia and attention deficit hyperactivity disorder. All 48 variants in M3 for which comprehensive testing was completed produce a gain-of-function (28/48) compared to loss-of-function (9/48); 11 variants had an indeterminant phenotype. This supports the idea that a key structural feature of the M3 gate exists to stabilize the closed state so that agonist binding can drive channel opening. Given that most M3 variants enhance channel gating, we assessed the potency of FDA-approved NMDAR channel blockers on these variant receptors. These data provide new insight into the structure–function relationship of the NMDAR gate, and suggest that variants within the M3 transmembrane helix produce a gain-of-function.

**Supplementary Information:**

The online version contains supplementary material available at 10.1007/s00018-023-05069-z.

## Introduction

N-methyl-d-aspartate receptors (NMDARs) are ligand-gated ion channels that mediate a slow Ca^2+^-permeable component of the excitatory synaptic current throughout the central nervous system [1]. NMDARs not only play an essential role in development, synaptic plasticity, and learning, but also are linked to a wide spectrum of neurological and psychiatric disorders such as epilepsy, Parkinson’s disease, autism, and schizophrenia [1–5]. NMDA receptors have also been considered potential targets to improve symptoms in a number of conditions, notably depression [6, 7]. The NMDARs are encoded by the *GRIN* gene family, which includes *GRIN1*, *GRIN2A*-*D* and *GRIN3A-B*. Functional NMDARs are heterotetramers comprising two glycine-binding GluN1 and two glutamate-binding GluN2 subunits. All NMDAR subunits share a similar structural arrangement, which contains an extracellular amino-terminal domain (NTD, also known as ATD), an agonist binding domain (ABD, also known as LBD, ligand binding domain), a pore-forming transmembrane domain (TMD) comprising three transmembrane helices (M1, M3, M4) and a re-entrant loop (M2), and an intracellular carboxyl-terminal domain (CTD) [1].

Recent advances in next-generation whole exome sequencing technology have led to the identification of a multitude of clinically relevant de novo and rare variants in *GRIN* genes in patients with neurological or psychiatric disorders, including epilepsy, intellectual disability (ID), developmental delay, and schizophrenia [1, 8, 9]. These rare variants are distributed across *GRIN1, GRIN2A, GRIN2B*, and *GRIN2D* and alter amino acid residues located in all four semi-autonomous domains (NTD, ABD, TMD, and CTD), with an enrichment in the ABD and the TMD. Regional analysis of missense intolerance of GluN1, GluN2A and GluN2B subunits in the healthy population showed that some sub-regions of ABD and TMD have more severe missense depletion, which suggests that genetic variation in these regions is more likely to be associated with diseases [10]. For example, the M3 transmembrane helix, including the highly conserved SYTANLAAF motif, plays an essential role in channel gating and is virtually intolerant to variation. Although pathogenic missense variants affecting this region are involved in a series of neurological and neurodevelopmental disorders, data describing the functional consequences of these variants is limited [11, 12]. To address this mismatch in terms of variants and function, we report the pharmacological and functional properties of 48 missense variants located in M3 transmembrane helix from 56 patients. We use these and other available data to assess the overall effect of these variants using a recently described comprehensive strategy to classify variants as gain-of-function (GoF) or loss-of-function (LoF) [13]. We find that variants in this region that allow surface expression are more likely to produce a gain-of-function, raising the idea that the sequence of the M3 gate was selected to stabilize the channel closed state, which is a pre-requisite for ligand-based activation.

## Materials and methods

### Consent and study approval

This study was approved by the Medical Ethics Committee and the Institutional Review Boards of University of Colorado School of Medicine and Children's Hospital Colorado (COMIRB 16–1520), Federico II University, Brown University, Duke University, Emory University, University of Melbourne, Seattle Children’s Research Institute, Cedars-Sinai Medical Center, University of Pennsylvania, and University of Leipzig Hospitals and Clinics. All data of this study were analyzed anonymously.

All in vitro studies were conducted according to the guidelines of Emory University.

### Molecular biology

We studied recombinant cDNA encoding human GluN1-1a (referred to as GluN1; NCBI Reference Sequence NM_007327.3), GluN2A (NM_000833.4), and GluN2B (NM_000834.4). Site-directed mutagenesis was performed on complementary DNA (cDNA) encoding the human *GRIN* genes [14] using the QuikChange protocol with Pfu DNA polymerase (Stratagene La Jolla, CA, USA) to replicate the parental DNA strand with the desired mismatch incorporated into the primer. Methylated parental DNA was digested with Dpn I for 1 h at 37˚C and the nicked mutant DNA was transformed into TOP10 Competent Cells (Life Tech, Grand Islands, NY, USA). Bacteria were spun down and plasmid DNA isolated using the Qiagen Qiaprep Spin Miniprep kit (Hilden, Germany). Sequences were verified through the mutated region using dideoxy DNA sequencing (Eurofins MWG Operon, Huntsville, AL, USA). The plasmid vector hosting wild type (WT) human GluN1, human GluN2A, and human GluN2B was pCI-neo [15].

The cDNA was linearized using FastDigest (Thermo, Waltham, MA) restriction digestion at 37 °C for 1 h. Complementary RNA (cRNA) was synthesized in vitro from linearized wild type and mutant cDNA using the mMessage mMachine T7 kit (Ambion, Austin, TX, USA). Unfertilized *Xenopus laevis* stage VI oocytes were prepared from commercially available ovaries (Xenopus one Inc, Dexter, MI, USA). The ovary was digested with Collagenase Type 4 (Worthington-Biochem, Lakewood, NJ, USA) solution (850 μg/ml, 15 ml for a half ovary) in Ca^2+^-free Barth’s solution, which contained (in mM) 88 NaCl, 2.4 NaHCO_3_, 1 KCl, 0.82 MgSO_4_, 10 HEPES (pH 7.4 with NaOH) supplemented with 100 μg/mL gentamycin, 40 μg/mL streptomycin. The ovary was incubated in enzyme with gentle mixing at RT (room temperature, 23 ℃) for 2 h. The oocytes were rinsed 5 times with Ca^2+^-free Barth’s solution (35–40 ml of fresh solution each time) for 10 min each time, and further rinsed 4 more times with normal Barth’s solution (containing 0.33 Ca(NO_3_)_2_, 35–40 ml of fresh solution) on the mixer for 10 min each time. The sorted oocytes were kept at 16 ℃ in an incubator for future use. *Xenopus laevis* oocytes were injected with a 1:1 ratio of GluN1:GluN2A and GluN1:GluN2B cRNA that by weight was a total of 0.25–25 ng in 50 nL of RNAase-free water per oocyte [14]. Injected oocytes were maintained in normal Barth’s solution at 15–19 ℃.

### Two-electrode voltage clamp current recordings

Two-electrode voltage clamp (TEVC) current recordings were performed one to three days post-injection at room temperature (23˚C) as previously described [14, 16]. The extracellular recording solution contained (in mM) 90 NaCl, 1 KCl, 10 HEPES, 0.5 BaCl_2_, and 0.01 EDTA (pH7.4 with NaOH). Solution exchange was computer controlled through an 8-valve positioner (Digital MVP Valve, Hamilton, CT, USA). Oocytes were placed in a dual track chamber that shared a single perfusion line, allowing simultaneous recording from two oocytes. All concentration–response solutions were made in the extracellular recording solution. Voltage control and data acquisition were achieved by a two-electrode voltage-clamp amplifier (OC725C, Warner Instruments, Hamden, CT, USA). The voltage electrode was filled with 0.3 M KCl and the current electrode with 3 M KCl. Oocytes were held under voltage clamp at a holding potential of –40 mV unless otherwise indicated. MTSEA (2-aminoethyl methanethiol sulfonate hydrobromide, Toronto Research Chemicals, Ontario, Canada) solution was prepared fresh and used within 30 min. All chemicals were obtained from Sigma–Aldrich unless otherwise stated.

*Xenopus* oocytes express a Ca^2+^-activated Cl^−^ current that confounds voltage clamp recordings of NMDAR-mediated currents in the presence of extracellular Ca^2+^. We, therefore, substituted Ba^2+^ for Ca^2+^, because Ba^2+^ permeates NMDARs and is relatively ineffective at the Ca^2+^-activated Cl^−^ current. Potential changes in the permeability to the divalent cation Ba^2+^ for WT and variant NMDARs were assessed by recording the current–voltage relationship in normal external recording solution (described above) and then again in external recording solution in which 90 mM NaCl was replaced with 60 mM BaCl_2_. The current–voltage relationship was determined from the difference in currents recorded during 15 mV step changes in the holding potential from 0 mV to − 90, − 75, − 60, − 45, − 30, − 15, 0, + 15, and + 30 mV before and during application of 100 µM glutamate plus 100 µM glycine for Na^+^ and for Ba^2+^ as the charge carrier in the same oocyte. Substitution of BaCl_2_ for NaCl was predicted to produce a − 6 mV shift in junction potential (pClamp, Molecular Devices), which was not corrected. The shift in the reversal potential between Ba^2+^ and Na^+^ solutions was determined for WT (∆V_REV,__WT_) and for variant (∆V_REV__,__VARIANT_) NMDARs, and then the differences between variant and WT reversal potential shifts was determined as ∆∆V_REV_ = (∆V_REV,VARIANT_ – ∆V_REV,WT_). The mean ± SEM for ∆V_REV_ for WT GluN1/GluN2A NMDARs was 12.1 ± 1.3 mV (*n* = 15), which corresponds to a 99% confidence interval of ± 3.4 mV. We, therefore, considered WT and variant reversal potential shifts in ∆V_REV_ that were greater than ± 3.4 mV as evidence for potential changes in Ba^2+^ permeability, which we assume is reflective of Ca^2+^ permeability.

### Cell culture and transfection

HEK293 cells (hereafter HEK cells; CRL 1573, ATCC, Manassas, VA, USA) for di-heteromeric receptor experiments were maintained in standard DMEM/Gluta-Max media (Fisher) with 10% dialyzed fetal bovine serum (R&D Systems) plus 10 U/ml and 10 µg/ml streptomycin (Fisher) at 37 ℃ and 5% CO_2_. The calcium phosphate method was used to transiently transfect the cells with plasmid cDNA encoding green fluorescent protein (GFP) and NMDAR GluN subunits in pcIneo (5:1:1 for GFP:GluN1:GluN2A or variant GluN2A, 1:1:1 for GFP:GluN1:GluN2B or variant GluN2B), as previously described [17]. Each well was transfected with 12.5 mM CaCl_2_, 2.5 mM BES solution (N,N-Bis(2-hydroxyethyl-2-aminethanesulfonic acid, N,N-Bis(2-hydroxyethyl)taurine; 14 mM NaCl; 75 µM Na_2_HPO_4_, pH 6.95) and 1 µg/ml of a DNA mixture for 4 h at 37 ℃, then washed and incubated overnight in standard media. NMDAR antagonists APV (d,l-2-amino-5-phosphonovalerate, 200 µM) and 7-CKA (7-chlorokynurenic acid, 200 µM) were added to reduce cell death caused by excessive activation of NMDARs.

### Whole cell voltage clamp recording

Whole cell voltage clamp recordings were performed on transiently transfected HEK cells 12–72 h post transfection. The patch electrodes (resistance 3–5 MΩ) for whole cell voltage clamp current recordings were pulled from thin-walled glass micropipettes (TW150F-4, World Precision Instruments, Sarasota, FL, USA) by a dual-stage glass micropipette puller (PC-10, Narishige, Tokyo, Japan) and filled with internal solution (in mM) 110 D-gluconate, 110 CsOH, 30 CsCl, 5 HEPES, 4 NaCl, 0.5 CaCl_2_, 2 MgCl_2_, 5 BAPTA, 2 NaATP and 0.3 NaGTP, pH 7.35. Transfected HEK cells were perfused with external recording solution that contained (in mM) 150 NaCl, 10 HEPES, 22 D-mannitol, 3 KCl, 1 CaCl_2_, and 0.01 EDTA (pH 7.4, 230C). The current response was recorded with an Axopatch 200B amplifier (Molecular Devices, Union City, CA, USA) at a holding potential of − 60 mV at room temperature (23 ℃). A two-barreled theta-glass micropipette was used for rapid solution exchange controlled by a piezoelectric translator (Burleigh Instruments, Newton, NJ, USA).

### Beta-lactamase assay

HEK cells were plated in 96-well plates (50,000 cells per well) and transiently transfected using Fugene6 with cDNA encoding β-lac-GluN1 variants and wild type GluN2, or wild type GluN1 and β-lac-GluN2 variants (Promega, Madison, WI) [10]. Cells treated with Fugene6 alone were used to define background absorbance. NMDAR antagonists (200 μM APV and 200 μM 7-CKA) were added at the time of transfection. Six wells were transfected for each condition; surface and total protein levels were measured in three wells each. After 24 h, cells were rinsed with Hank’s Balanced Salt Solution (HBSS, in mM, 140 NaCl, 5 KCl, 0.3 Na_2_HPO_4_, 0.4 KH_2_PO_4_, 6 glucose, 4 NaHCO_3_) supplemented with 10 mM HEPES, and then in HBSS/HEPES solution supplemented with 100 μM nitrocefin (100 μL; Millipore, Burlington, MA, USA) solution to each well for measuring the level of extracellular enzymatic activity, which reflected NMDAR surface expression. To determine the level of total enzymatic activity, the cells were lysed by a 30 min incubation in 50 μL H_2_O prior to the addition of 50 μL of 200 μM nitrocefin in HBSS/HEPES. The absorbance at 486 nm was read using a microplate reader every min for 30 min at 30 ℃. The rate of increase in absorbance was generated from the slope of a linear fit to the data.

### Evaluation of FDA-approved NMDAR inhibitors

FDA-approved drugs that act as NMDAR open channel blockers (memantine, ketamine, dextromethorphan and its metabolite dextrorphan) were evaluated using TEVC recordings from *Xenopus* oocytes co-expressing GluN1 variants with the WT GluN2, and WT GluN1 with GluN2 variants. The composite concentration–response curves were recorded at a holding potential of − 40 mV and fitted to determine the IC_50_ values (*see* below).

### Data and statistical analysis

Statistical analyses were performed in GraphPad Prism 8.0.1 (La Jolla, CA, USA) and OriginPro 9.0 (Northampton, MA, USA). Statistical significance was assessed using one-way ANOVA with Post hoc Dunnett’s Multiple Comparison Test, with *p* < 0.05 considered significant. Power was determined using GPower (3.1.9.2). Data are presented as mean ± SEM. Error bars represent SEM unless otherwise stated. The concentration–response relationship for agonists were fitted by Eq. 1, and the concentration–response relationship for inhibition by FDA-approved channel blockers was fitted by Eq. 2,

1 $${\text{Response}}\left( \% \right) = \, 100\% \, / \, \left( {1 \, + \, \left( { \, EC_{50} / \, \left[ {{\text{agonist}}} \right] \, } \right)^{N} } \right),$$

2 $${\text{Response}}\left( \% \right) = \, \left( {100\% \, - {\text{minimum}}} \right) \, / \, \left( {1 \, + \, \left( { \, \left[ {{\text{concentration}}} \right] \, / \, IC_{50} } \right)^{N} } \right) \, + {\text{minimum}}$$

where* N* is the Hill slope, EC_50_ is the concentration of the agonist that produces a half-maximal effect, IC_50_ is the concentration of the inhibitor that produces a half-maximal effect, *minimum* is the degree of residual response at a saturating concentration of the inhibitor, constrained during fitting to be > 0, and *Response* is expressed as a percent of the fitted maximum. The maximal channel open probability (*P*_*OPEN*_) when activated by maximally effective concentrations of agonist was estimated from the fold *Potentiation* observed in MTSEA using Eq. 3 [18]:

3 $$P_{OPEN} = \, \left( {\gamma_{MTSEA} /\gamma_{CONTROL} } \right) \, \times \, \left( {1 \, /Potentiation} \right)$$

where *γ*_*MTSEA*_ and *γ*_*CONTROL*_ were the single channel chord conductance values estimated from GluN1/GluN2A receptors and fold *Potentiation* was defined as the ratio of current in the presence of MTSEA to current in the absence of MTSEA, and *γ*_*MTSEA*_ / *γ*_*CONTROL*_ was 0.67 [18]. Rise time for each response was determined as the time measured between 10 and 90% of the peak current. The current response time course was fitted using ChanneLab (Synaptosoft, Decatur, GA, USA) by Eq. 4*,*

4 $$Current\left( {pA} \right) = Amplitude_{FAST} \exp \left( { - time/ \, \tau_{FAST} } \right) \, + Amplitude_{SLOW} \exp \left( { - time/ \, \tau_{SLOW} } \right)$$

The weighted deactivation tau (τ_w_) was calculated by Eq. 5*,*

5 $$\tau_{w} = \, (Amplitude_{FAST} \tau_{FAST} + Amplitude_{SLOW} \tau_{SLOW} )/\left( {Amplitude_{FAST} + Amplitude_{SLOW} } \right)$$

Synaptic charge transfer was estimated as the product of peak whole cell current response amplitude and the weighted deactivation tau (τ_w_) for the termination of responses following rapid removal of glutamate. We calculated the relative fold-change in synaptic charge transfer with Eq. 6 [13]:

6 $${\text{Charge transfer}}_{{{\text{Synaptic}}}} = \, \tau_{{{\text{wMUT}}}} /\tau_{{{\text{wWT}}}} \times P_{{{\text{MUT}}}} /P_{{{\text{WT}}}} \times Surf_{{{\text{MUT}}}} /Surf_{{{\text{WT}}}} \times R_{{{\text{GLY}}}} \times R_{{{\text{GLU}},{\text{ Synaptic}}}} \times Mg_{{{\text{MUT}}}} /Mg_{{{\text{WT}}}}$$

where τ_w_ is the mean weighted deactivation time constant, *P* is the receptor maximum open probability, *Surf* is surface protein levels, *Mg* is percentage inhibition by 1 mM Mg^2+^ at a holding potential between – 40 and – 60 mV, and *R*_GLY_ and *R*_GLU, Synaptic_ are a ratio of the relative response for variant to WT NMDARs to an extracellular concentration of glycine assumed to be 3 × 10^−6^ M and a synaptic concentration of 1 × 10^−3^ M glutamate, respectively.

We calculated the relative fold-change in non-synaptic charge transfer in a similar manner, with time dependence removed and 1 × 10^−7^ M glutamate for R_GLU, Non-synaptic_ by Eq. 7 [13]:

7 $${\text{Charge transfer}}_{{{\text{Non}} - {\text{synaptic}}}} = P_{{{\text{MUT}}}} /P_{{{\text{WT}}}} \times Surf_{{{\text{MUT}}}} /Surf_{{{\text{WT}}}} \times R_{{{\text{GLY}}}} \times R_{{{\text{GLU}},{\text{ Non}} - {\text{Synaptic}}}} \times Mg_{{{\text{MUT}}}} /Mg_{{{\text{WT}}}}$$

We classified variants as gain-of-function (GoF) or loss-of-function (LoF) according to criteria described in Myers et al. [13]. For some variants in which we executed all six assays, the current amplitude of NMDAR responses in transfected HEK cells recorded under voltage clamp was too small to accurately determine the deactivation tau, and thus these variants were classified as Indeterminant* given we had data in all other assays but cannot determine the overall outcome. The small amplitude could be the result of altered receptor function leading to cell death or detachment of cells with a large number of NMDARs on the surface. In addition, some variants that were initially deemed as a conflict according to Myers et al. [13] were considered *Indeterminant* when the synaptic and non-synaptic charge transfers were both suprathreshold but in conflict.

### Three-dimensional Missense tolerance determination

The three-dimensional missense tolerance ratios (3DMTR, [19]) of the diheteromeric NMDARs were calculated using homology models based on the non-active GluN1/GluN2B structure (pdb: 6WHS, [20]), using the 3DMTR application (https://github.com/riley-perszyk/3DMTR). In this analysis, the non-neuro gnomAD (v.2.1.1) dataset was used. The 3DMTR assesses the intolerance of each residue as a running average using the central residue along with the intolerance of the 30 closest residues in 3-dimensional space, determined from the structure of the receptor [19].

## Results

### Variants in M3 transmembrane helix and neurological and neurodevelopmental disorders

We identified 48 variants in the M3 transmembrane helix across *GRIN1, GRIN2A*, and *GRIN2B* in 56 patients with neurological and/or neurodevelopmental disorders (Table 1, Supplemental Table S1). 22 of these variants were reported in the peer-reviewed literature or ClinVar (https://www.ncbi.nlm.nih.gov/clinvar/) and others were newly discovered. According to the clinical data from 47 patients (*see* Supplemental Table S1), the most common phenotypes of these patients with M3 transmembrane variants are epilepsy (70%, 33/47), intellectual disability (70%, 33/47), developmental delay (51%, 24/47), movement disorders (28%, 13/47), language disorders (15%, 7/47), and autism spectrum disorder (ASD; 6.4%, 3/47). Clinical information for the remaining 9 patients was not available.

**Table 1:** Information of patients and *GRIN* variants

	Gene	Variant	Genotype	Protein	Origin	Phenotype	Source
1	*GRIN1*	GluN1-A637S	c.1909G > T	p.Ala637Ser	de novo	DD, ID	This study
2	*GRIN1*	GluN1-A637V	c.1910C > T	p.Ala637Val	de novo	Epi, DD, ID, autism	This study, ClinVar
3	*GRIN1*	GluN1-G638A	c.1913G > C	p.Gly638Ala	n.a	ID	This study, ClinVar
4	*GRIN1*	GluN1-G638V	c.1913G > T	p.Gly638Val	de novo	Epi, DD, IS	This study
5	*GRIN1*	GluN1-M641I	c.1923G > A	p.Met641Ile	de novo	Epi, MD, ID	This study, [43, 53]
6	*GRIN1*	GluN1-M641L	c.1921A > T	p.Met641Leu	de novo	Epi, EOEE, MD, ID	This study, [54], ClinVar
7	*GRIN1*	GluN1-M641V	c.1921A > G	p.Met641Val	de novo	DD, ID	This study, ClinVar
8	*GRIN1*	GluN1-I642L	c.1924A > C	p.Ile642Leu	n.a	n.a	This study, ClinVar
9	*GRIN1*	GluN1-I642T	c.1925 T > C	p.Ile642Thr	n.a	DD, MD	This study
10	*GRIN1*	GluN1-I643V	c.1927A > G	p.Ile643Val	n.a	DEE, ID	This study, ClinVar
11	*GRIN1*	GluN1-V644M	c.1930G > A	p.Val644Met	n.a	n.a	This study, ClinVar
12	*GRIN1*	GluN1-A645S	c.1933G > T	p.Ala645Ser	de novo	Epi, ID, CVI, ASD	[55], ClinVar
13	*GRIN1*	GluN1-Y647C	c.1940A > G	p.Tyr647Cys	de novo	Epi, ID, MD, CVI	[56]
14	*GRIN1*	GluN1-Y647S	c.1940A > C	p.Tyr647Ser	de novo	Epi, DD, IS	This study, [55, 57]
15	*GRIN1*	GluN1-N650I	c.1949A > T	p.Asn650Ile	de novo	Epi, ID	This study, ClinVar
16	*GRIN1*	GluN1-N650K	c.1950C > G	p.Asn650Lys	de novo	Epi, ID, DD, MD	[53], ClinVar
17	*GRIN1*	GluN1-A652T	c.1954G > A	p.Ala652Thr	de novo	Epi, ID, LD	This study
18	*GRIN1*	GluN1-A653G	c.1958C > G	p.Ala653Gly	de novo	n.a	[55], ClinVar
19	*GRIN1*	GluN1-A653T	c.1957G > A	p.Ala653Thr	n.a	DD	[58]
20	*GRIN1*	GluN1-F654C	c.1961 T > G	p.Phe654Cys	de novo	Epi, EE, ID	This study, ClinVar
21	*GRIN1*	GluN1-L655Q	c.1964 T > A	p.Leu655Gln	de novo	Epi, ID, DD	This study, ClinVar
22	*GRIN2A*	GluN2A-S632F	c.1895C > T	p.Ser632Phe	n.a	n.a	This study, ClinVar
23	*GRIN2A*	GluN2A-A635T	c.1903G > A	p.Ala635Thr	de novo	Epi, ID, DD, LD, MD	This study, [59], ClinVar
24	*GRIN2A*	GluN2A-V639I	c.1915G > A	p.Val639Ile	n.a	Epi	This study, ClinVar
25	*GRIN2A*	GluN2A-L642M	c.1924C > A	p.Leu642Met	de novo	DD, ID	This study, ClinVar
26	*GRIN2A*	GluN2A-L642R	c.1925 T > G	p.Leu642Arg	de novo	Epi, ID, MD	[60], ClinVar
27	*GRIN2A*	GluN2A-A643D	c.1928C > A	p.Ala643Asp	de novo	ID, DD, MD, LD	[61], ClinVar
28	*GRIN2A*	GluN2A-S644G	c.1930A > G	p.Ser644Gly	de novo	Epi, ID, DD, CVI	[48], ClinVar
29	*GRIN2A*	GluN2A-T646A	c.1936A > G	p.Thr646Ala	de novo	Epi, ID, DD, LD, MD	This study, [59, 62] ClinVar
30	*GRIN2A*	GluN2A-T646R	c.1937C > G	p.Thr646Arg	n.a	n.a	This study, ClinVar
31	*GRIN2A*	GluN2A-N648S	c.1943A > G	p.Asn648Ser	de novo	Epi, ID	This study, [59, 63], ClinVar
32	*GRIN2A*	GluN2A-L649V	c.1945C > G	p.Leu649Val	de novo	Epi, ID, DD, CVI	[59, 64] ClinVar
33	*GRIN2A*	GluN2A-A650S	c.1948G > T	p.Ala650Ser	de novo	Epi, DD	This study, ClinVar
34	*GRIN2A*	GluN2A-F652V	c.1954 T > G	p.Phe652Val	de novo	Epi, DD, ID, LD, MD, ASD	[65], ClinVar
35	*GRIN2A*	GluN2A-M653I	c.1959G > A	p.Met653Ile	de novo	Epi, DD, ID	[59], ClinVar
36	*GRIN2A*	GluN2A-M653V	c.1957A > G	p.Met653Val	de novo	Epi, DD, ID	[59], ClinVar
37	*GRIN2A*	GluN2A-I654T	c.1961 T > C	p.Ile654Thr	de novo	Epi, ID, DD, LD, MD	[59], ClinVar
38	*GRIN2B*	GluN2B-A636P	c.1906G > C	p.Ala636Pro	de novo	ID, DD, ADHD	[66, 67] ClinVar
39	*GRIN2B*	GluN2B-A636V	c.1907C > T	p.Ala636Val	de novo	Epi, ID, MCD, CVI	[66], ClinVar
40	*GRIN2B*	GluN2B-A639V	c.1916C > T	p.Ala639Val	de novo	Epi, ID, MCD, CVI	[66, 68, 69], ClinVar
41	*GRIN2B*	GluN2B-I641T	c.1922 T > C	p.Ile641Thr	n.a	n.a	This study, ClinVar
42	*GRIN2B*	GluN2B-Y646C	c.1937A > G	p.Tyr646Cys	de novo	DEE	This study, ClinVar
43	*GRIN2B*	GluN2B-N649S	c.1946A > G	p.Asn649Ser	n.a	DEE	This study, ClinVar
44	*GRIN2B*	GluN2B-N649T	c.1946A > C	p.Asn649Thr	n.a	n.a	This study, ClinVar
45	*GRIN2B*	GluN2B-A652P	c.1954G > C	p.Ala652Pro	n.a	DD, ID, MD	This study, ClinVar
46	*GRIN2B*	GluN2B-A652G	c.1955C > G	p.Ala652Gly	n.a	n.a	This study, ClinVar
47	*GRIN2B*	GluN2B-F653V	c.1957 T > G	p.Phe653Val	n.a	DD, MD	This study
48	*GRIN2B*	GluN2B-I655F	c.1963A > T	p.Ile655Phe	de novo	Epi, ID, MCD, MC, CVI	[66], ClinVar

*ADHD* Attention deficit hyperactivity disorder, *ASD* Autism spectrum disorder, *CVI* Cerebral visual impairment, *DD* Developmental delay, *DEE* Developmental and epileptic encephalopathy, *EOEE* Early-onset epileptic encephalopathies, *Epi* epilepsy/seizures, *ID* Intellectual disability, *IS* infantile spams, *LD* language disorder (including language delay, speech disorders), *MCD* Malformations of cortical development, *MD* movement disorder, *n.a.* not available or unknown

More detailed clinical information is presented in Supplemental Table S1

**Fig. 1:**
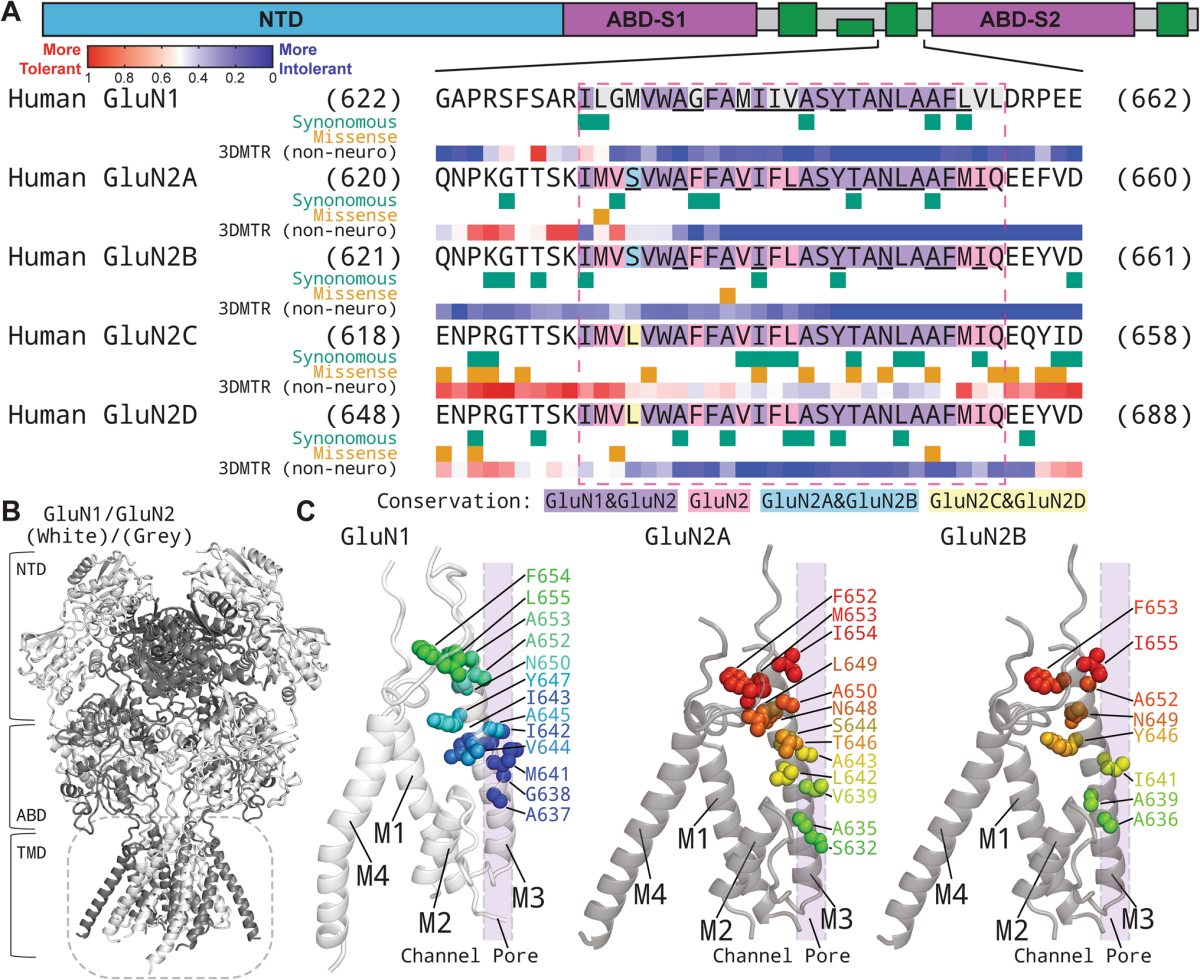
Locations of disease-associated missense variants in the M3 transmembrane helix. **A** A linear schematic showing domain architecture of the *GRIN*/GluN and protein residue sequence alignment for highly conserved M3 transmembrane domain (TMD) across GluN1 and GluN2 subunits. NTD indicates the N-terminal domain (also known as ATD, amino terminal domain); S1 and S2 denote the first and second polypeptide sequences comprising the agonist binding domain (ABD). The aligned amino-acid sequence of the M3 helix of each NMDAR subunits is listed with a raster plot depicting the missense and synonymous variants found in gnomAD along with the 3DMTR score for these stretches, shown as a colorimetric raster plot. The red dashed box indicates the M3 transmembrane helix. **B** Homology model of the GluN1/GluN2A receptor built from previously reported GluN1/GluN2B cryo-EM data [20]. **C** The residues harboring missense variants are highlighted with different colors in a side view; the side of the subunit facing the pore is indicated

The residues in the M3 transmembrane helix are highly conserved in different subunits within the *GRIN*/GluN family (Fig. 1A) and contain the SYTANLAAF motif that is largely invariant throughout the glutamate receptor family. The intolerance of M3 to genetic variation was assessed with the 3D Missense Tolerance Ratio [19] across *GRIN1, GRIN2A, GRIN2B, GRIN2C* and *GRIN2D* genes, which revealed minimal regional variation for M3 for all subunits except GluN2C (Fig. 1A). This result is consistent with strong purifying selection [21] acting upon much of the M3 transmembrane helix and suggests that this region is highly intolerant to genetic variation. The variants studied here all fall into the intolerant region, and span the full M3 transmembrane domain, as shown in Fig. 1B, C.

### Variants in the M3 transmembrane helix alter agonist potency

**Fig. 2:**
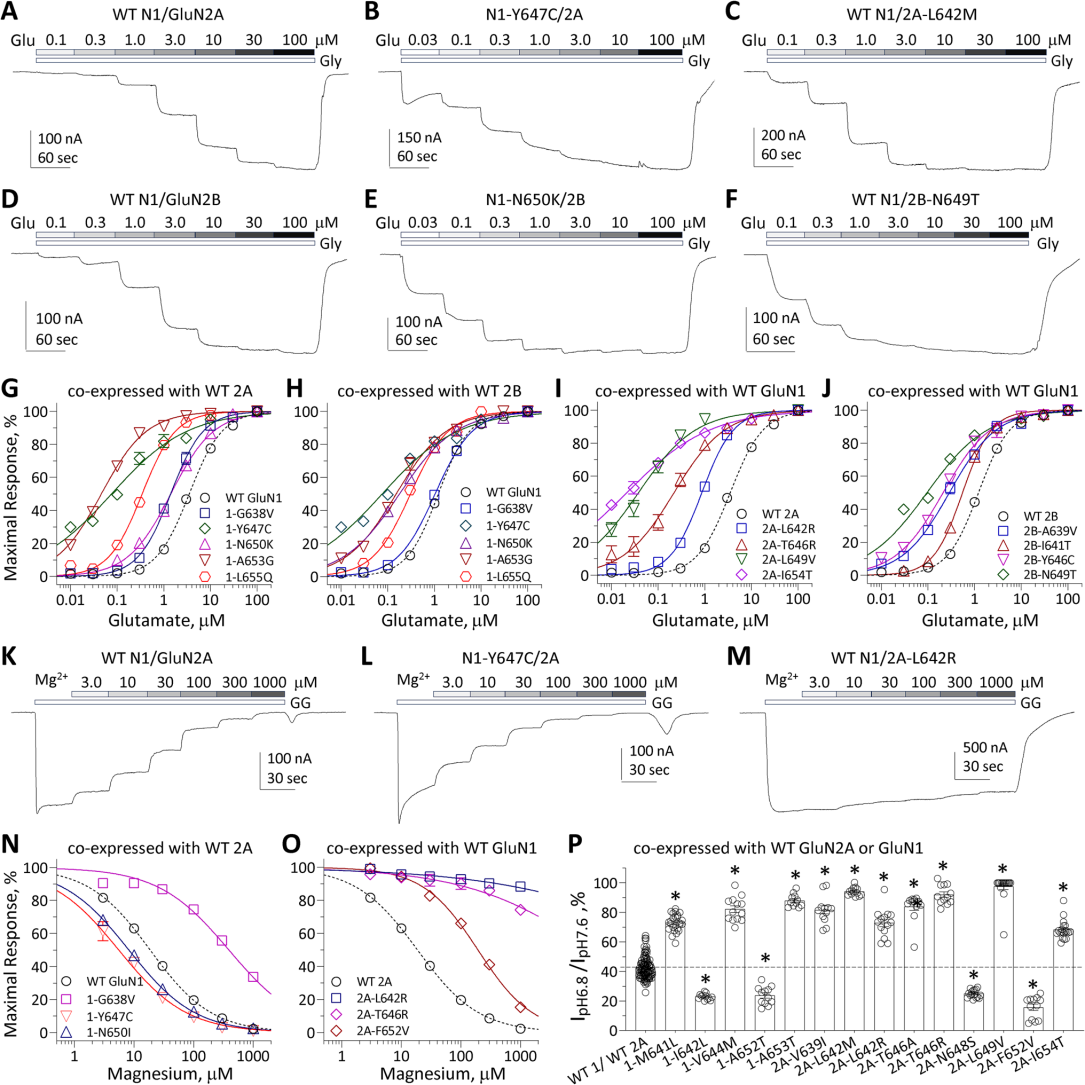
Variants in the M3 transmembrane helix influence pharmacological and biophysical properties of NMDARs. **A–F** Representative two electrode voltage clamp current recordings from *Xenopus* oocytes expressing GluN1/GluN2A and GluN1/GluN2B wild type or variant NMDAR subunits, as indicated. The glutamate concentration–response relationship was determined by co-applying increasing concentrations of glutamate with maximally effective concentration of glycine (30-100 µM). **G–J** Composite concentration–response curves for glutamate in the presence of 30-100 µM glycine fitted with the Hill equation (*see* Methods). **K–M** Representative voltage clamp current recordings show the Mg^2+^ concentration–response relationship for GluN1/GluN2A wild type and variants (as indicated) determined by co-applying increasing concentrations of Mg^2+^ with 100 µM glutamate and 100 µM glycine. **N**, **O** Composite concentration–response curves are shown for Mg^2+^ inhibition recorded at a holding potential of –60 mV for wild type and variant GluN1/GluN2A. **P** Summary of the effects of GluN1 and GluN2A variants on proton sensitivity, evaluated by the ratio of current response at pH 6.8 to pH 7.6 at a holding potential of − 40 mV

Two-electrode voltage clamp current recordings in *Xenopus laevis* oocytes expressing GluN1 variants with wild type (WT) GluN2A or GluN2B, and WT GluN1 with GluN2A or GluN2B variants were performed to evaluate the functional effects of variants in the M3 transmembrane helix. The half-maximally effective concentration (EC_50_) of agonists was determined by analyzing the glutamate and glycine concentration-effect relationships. Almost all M3 variant-containing NMDARs (37/48) showed a significantly increased glutamate and glycine potency (i.e., decreased EC_50_ values) including GluN1-A652T which increased glutamate potency when expressed only with GluN2B. Only 3/48 variants decreased glutamate potency by ~ twofold or less, 6/48 variants had no detectable effect on EC_50_ (Table 2), and 2/48 variants produced responses that were too small to measure. Some M3 variant-containing NMDARs exhibited over a 100-fold increase in potency compared to WT NMDARs, and a number of variants produced more than a tenfold increase glutamate potency (reduced EC_50_ values), including GluN1-V644M, GluN1-Y647C, GluN1-Y647S, GluN1-N650I, GluN1-A653G, GluN1-A653T, GluN1-F654C, GluN1-L655Q, GluN2A-S644G, GluN2A-T646A, GluN2A-T646R, GluN2A-L649V, GluN2A-M653I, GluN2A-M653V, GluN2A-I654T, GluN2B-N649S, and GluN2B-N649T (Fig. 2A–J, Table 2). One potential interpretation of these data is that the M3 transmembrane helix is invariant to enable the NMDAR to be activated by micromolar concentrations of glutamate, as virtually any change in the amino acid side chain is not tolerated and usually lowers glutamate EC_50_ values. This is consistent with the idea that the invariant SYTANLAAF region of the M3 transmembrane helix forms the gate that occludes the pore in the closed conformation. Figure 1C shows the location of these variants on an expanded cut-away view of the NMDAR pore.

**Table 2:** Pharmacological properties and receptor surface expression of variant NMDARs

	Glu EC_50_, µM (*n*)	Gly EC_50_, µM (*n*)	Mg^2+^ IC_50_, µM (*n*)	%, pH_6.8_/pH_7.6_	Surface/total Ratio
WT GluN1/2A	3.5 [3.4, 3.6] (113)	1.2 [1.1, 1.2] (122)	19 [17, 20] (113)	43 ± 0.7 (118)	1.0 (36)
1-A637S/2A	3.9 [3.1, 4.8] (12)	0.74 [0.67, 0.82] (12)*	48 [41, 55] (11)*	50 ± 1.9 (11)	0.87 ± 0.44 (3)
1-A637V/2A	2.8 [2.3, 3.2] (12)*	1.0 [0.88, 1.2] (12)	347 [257, 469] (12)*	39 ± 1.6 (12)	1.3 ± 0.09 (4)^δ^
1-G638A/2A	2.4 [2.1, 2.7] (12)*	0.88 [0.80, 0.96] (14)*	46 [34, 62] (11)*	53 ± 8.1 (12)^#^	1.4 ± 0.12 (4)^δ^
1-G638V/2A	1.3 [1.2, 1.4] (12)*	0.48 [0.43, 0.54] (12)*	435 [363, 522] (18)*	52 ± 1.4 (16)^#^	0.47 ± 0.12 (5)^δ^
1-M641I/2A^a,b^	3.4 [2.6, 4.2] (15)	1.1 [1.0, 1.2] (19)	161 [118, 204] (19)*	41 ± 0.22 (22)	0.71 ± 0.10 (5)^δ^
1-M641L/2A	1.2 [0.82, 1.9] (17)*	0.58 [0.49, 0.69] (18)*	13 [9.6, 18] (14)	73 ± 1.2 (25)^#^	0.88 ± 0.08 (4)
1-M641V/2A	2.5 [2.2, 2.9] (12)*	0.85 [0.73, 0.99] (12)*	31 [27, 35] (12)*	50 ± 1.2 (12)^#^	1.3 ± 0.11 (6)
1-I642L/2A	8.6 [8.0, 9.1] (12)*	2.0 [1.9, 2.2] (12)*	21 [19, 23] (12)	21 ± 1.6 (12)^#^	0.78 ± 0.09 (4)^δ^
1-I642T/2A	7.1 [6.5, 7.7] (12)*	2.3 [2.1, 2.5] (12)*	17 [13, 21] (12)	35 ± 1.6 (12)^#^	1.8 ± 0.55 (3)
1-I643V/2A	1.9 [1.7, 2.1] (12)*	0.73 [0.63, 0.86] (12)*	23 [16, 32] (12)	60 ± 2.4 (12)^#^	0.84 ± 0.03 (6)
1-V644M/2A	0.30 [0.25, 0.36] (12)*	0.14 [0.11, 0.18] (12)*	38 [31, 46] (14)*	82 ± 2.3 (12)^#^	1.0 ± 0.09 (4)
1-A645S/2A	3.2 [3.0, 3.5] (18)	1.0 [0.88, 1.1] (15)	41 [32, 52] (12)*	62 ± 2.4 (24)^#^	0.79 ± 0.21 (5)
1-Y647C/2A	0.08 [0.05, 0.12] (16)*	0.023 [0.014, 0.037] (14)*	6.0 [5.2, 7.0] (23)*	34 ± 1.3 (31)^#^	0.24 ± 0.02 (4)^δ^
1-Y647S/2A	0.069 [0.05, 0.10] (15)*	0.043 [0.033, 0.058] (17)*	12 [6.7, 20] (5)	27 ± 1.1 (26)^#^	0.16 ± 0.05 (4)^δ^
1-N650I/2A	0.037 [0.026, 0.053] (12)*	0.046 [0.034, 0.062] (16)*	8.3 [6.5, 11] (12)*	47 ± 1.4 (12)	0.76 ± 0.12 (4)^δ^
1-N650K/2A	0.46 [0.36, 0.59] (11)*	0.22 [0.20, 0.24] (12)*	46 [40, 53] (14)*	39 ± 3.1 (12)	0.055 ± 0.012 (5)^δ^
1-A652T/2A	4.0 [3.4, 4.7] (24)	1.0 [0.95, 1.1] (14)	19 [15, 23] (18)	23 ± 1.2 (18)^#^	1.1 ± 0.16 (6)
1-A653G/2A	0.15 [0.12, 0.17] (11)*	0.12 [0.090, 0.14] (12)*	22 [18, 29] (13)	37 ± 3.0 (14)	0.023 ± 0.004 (6)^δ^
1-A653T/2A	0.12 [0.10, 0.14] (14)*	0.024 [0.019, 0.031] (12)*	23 [18, 30] (12)	88 ± 1.3 (11)^#^	1.1 ± 0.08 (4)
1-F654C/2A	0.26 [0.21, 0.32] (12)*	0.13 [0.11, 0.15] (12)*	22 [18, 27] (12)	46 ± 1.5 (12)	1.4 ± 0.13 (4)^δ^
1-L655Q/2A	0.34 [0.32, 0.35] (16)*	0.095 [0.080, 0.11] (15)*	18 [15, 20] (8)	46 ± 0.5 (12)	0.86 ± 0.04 (5)^δ^
WT GluN1/2A	3.5 [3.4, 3.6] (113)	1.2 [1.1, 1.2] (122)	19 [17, 20] (113)	43 ± 0.7 (118)	1.0 (42)
2A-S632F	3.6 [3.2, 4.0] (12)	1.4 [1.3, 1.5] (12)*	93 [74, 116] (11)*	37 ± 1.4 (12)	0.96 ± 0.157 (8)
2A-A635T	0.96 [0.83, 1.1] (12)*	0.28 [0.23, 0.33] (12)*	56 [42, 76] (12)*	60 ± 1.7(14)^#^	0.76 ± 0.26 (10)
2A-V639I	0.53 [0.46, 0.61] (14)*	0.26 [0.23, 0.31] (12)*	33 [27, 41] (12)*	82 ± 2.7(14)^#^	1.21 ± 0.26 (6)
2A-L642M	0.61 [0.43, 0.86] (13)*	0.36 [0.28, 0.47] (12)*	35 [27, 44] (12)*	94 ± 0.78 (13)^#^	0.37 ± 0.02 (4)^δ^
2A-L642R	0.87 [0.75, 1.0] (14)*	0.31 [0.28, 0.34] (14)*	> 1000 (13)*	73 ± 2.5 (14)^#^	0.10 ± 0.05 (4)^δ^
2A-A643D^c^	1.0 [0.70, 1.4] (23)*	0.11 [0.093, 0.13] (13)*	39 [28, 56] (12)*	48 ± 1.5 (13)	0.44 ± 0.06 (4)^δ^
2A-S644G^d^	0.17 [0.13, 0.22] (21)*	0.076 [0.047, 0.094] (14)*	33 [27, 35] (26)*	97 ± 1.2 (19)^#^	0.34 ± 0.10 (4)^δ^
2A-T646A	0.14 [0.08, 0.24] (12)*	0.071 [0.058, 0.087] (14)*	35 [26, 47] (12)*	84 ± 2.7 (12)^#^	0.041 ± 0.02 (5)^δ^
2A-T646R	0.21 [0.13, 0.34] (6)*	0.0068 [0.0026, 0.018] (6)*	> 1000 (6)*	92 ± 1.9 (12)^#^	0.91 ± 0.09 (6)
2A-N648S	1.3 [1.1, 1.4] (16)*	0.45 [0.36, 0.57] (12)*	15 [12, 18] (13)	25 ± 0.7 (16)^#^	0.27 ± 0.09 (6)^δ^
2A-L649V	0.035 [0.022, 0.056] (11)*	0.012 [0.0075, 0.020] (8)*	69 [58, 81] (8)*	97 ± 2.0 (18)^#^	0.34 ± 0.03 (4)^δ^
2A-A650S	2.8 [2.5, 3.1] (12)*	0.80 [0.70, 0.91] (12)*	41 [32, 53] (8)*	68 ± 1.5 (10)^#^	0.47 ± 0.16 (5)^δ^
2A-F652V	0.93 [0.78, 1.1] (12)*	0.65 [0.54, 0.78] (8)*	187 [154, 228] (16)*	16 ± 2.2 (12)^#^	0.86 ± 0.09 (4)
2A-M653I	0.17 [0.14, 0.20] (14)*	0.062 [0.048, 0.082] (13)*	11 [10, 13] (12)*	19 ± 1.2 (12)^#^	0.29 ± 0.03 (4)^δ^
2A-M653V	0.058 [0.040, 0.084] (19)*	0.055 [0.042, 0.071] (11)*	62 [44, 87] (17)*	32 ± 2.5 (27)^#^	0.51 ± 0.19 (3)^δ^
2A-I654T	0.016 [0.007, 0.034] (14)*	0.061 [0.052, 0.072] (12)*	30 [23, 38] (12)*	68 ± 1.5 (19)^#^	0.22 ± 0.06 (4)^δ^
WT GluN1/2B	1.2 [1.1, 1.2] (66)	0.39 [0.35, 0.44] (62)	22 [20, 25] (59)	16 ± 0.5 (93)	1.0 (10)
1-G638V/2B	0.92 [0.74, 1.1] (16)	0.20 [0.17, 0.23] (12)*	296 [150, 581] (7)*	13 ± 0.8 (30)	0.57 ± 0.11 (4)^δ^
1-M641I/2B^a^	1.5 (1.0, 2.1) (11)	0.27 [0.14, 0.41] (13)	408 [222, 594] (15)*	14 ± 0.9 (13)	1.1 ± 0.12 (6)
1-A645S/2B	1.6 [1.4, 1.8] (12)	0.45 [0.35, 0.59] (13)	67 [50, 90] (12)*	20 ± 1.2 (17)	1.0 ± 0.15 (6)
1-Y647C/2B	0.034 [0.02, 0.06] (9)*	0.0233 [0.014, 0.037] (14)*	14 [10, 20] (10)	30 ± 2.8 (12)^#^	0.58 ± 0.09 (4)^δ^
1-N650K/2B	0.052 [0.046, 0.059] (12)*	0.041 [0.030, 0.055] (8)*	33 [27, 39] (12)*	19 ± 0.6 (12)	0.35 ± 0.06 (4)^δ^
1-A652T/2B	0.61 [0.50, 0.74] (12)*	0.12 [0.10, 0.13] (8)*	31 [24, 41] (8)	16 ± 2.0 (12)	0.70 ± 0.10 (4)
1-A653G/2B	0.17 [0.13, 0.21] (24)*	0.14 [0.11, 0.18] (17)*	36 [26, 49] (16)*	15 ± 0.5 (20)	0.32 ± 0.01 (4)^δ^
1-L655Q/2B	0.28 [0.27, 0.30] (6)*	0.13 [0.10, 0.18] (11)*	24 [18, 34] (6)	33 ± 0.8 (12)^#^	0.63 ± 0.13 (4)*
WT GluN1/2B	1.2 [1.1, 1.2] (66)	0.39 [0.35, 0.44] (62)	22 [20, 25] (59)	16 ± 0.5 (93)	1.0 (38)
2B-A636P	n.d	n.d	n.d	n.d	0.068 ± 0.02 (4)^δ^
2B-A636V	0.49 [0.43, 0.57] (18)*	0.15 [0.14, 0.17] (14)*	156 [146, 167] (12)*	36 ± 1.6 (12)^#^	0.36 ± 0.08 (10)^δ^
2B-A639V	0.28 [0.21, 0.36] (12)*	0.065 [0.050, 0.086] (12)*	14 [12, 16] (12)*	58 ± 6.1 (12)^#^	0.13 ± 0.07 (4)^δ^
2B-I641T	0.57 [0.47, 0.70] (13)*	0.15 [0.13, 0.18] (12)*	21 [16, 28] (12)	25 ± 1.6 (12)^#^	0.48 ± 0.09 (4)^δ^
2B-Y646C	0.22 [0.17, 0.30] (14)*	0.020 [0.014, 0.028] (13)*	30 [27, 34] (12)*	23 ± 2.4 (12)	0.58 ± 0.10 (3)^δ^
2B-N649S	0.10 [0.071, 0.15] (6)*	0.047 [0.021, 0.10] (4)*	23 [21, 26] (10)	16 ± 0.9 (12)	0.97 ± 0.08 (4)
2B-N649T	0.10 [0.077, 0.14] (16)*	0.056 [0.043, 0.073] (13)*	29 [25, 33] (15)	14 ± 1.2 (15)	0.86 ± 0.11 (4)
2B-A652G	1.4 [1.1, 1.7] (15)	0.26 [0.21, 0.33] (12)*	63 [44, 82] (12)*	40 ± 0.9 (15)^#^	1.5 ± 0.30 (5)
2B-A652P	4.1 [3.5, 4.9] (15)*	0.96 [0.81, 1.1] (11)*	19 [13, 28] (12)	37 ± 3.2 (12)^#^	1.0 ± 0.10 (4)
2B-F653V	n.d	n.d	n.d	n.d	n.a
2B-I655F^e^	1.3 [1.1, 1.5] (20)	0.37 [0.32, 0.42] (28)	22 [18, 27] (24)	67 ± 2.5 (22)^#^	0.56 ± 0.09 (6)^δ^

The concentration–response relationship for glutamate was determined in the presence of 0.1 mM glycine, and the glycine concentration–response relationship was determined in the presence of 0.1 mM glutamate. Data shown are the mean IC_50_ or EC_50_ value with 95% confidence intervals determined from the LogEC_50_ or LogIC_50_ values

^*^Indicates 95% confidence intervals that are non-overlapping with WT GluN1/GluN2A- or GluN1/GluN2B-containing NMDARs. Data are mean ± SEM for current ratio at different pH values and for surface/total ratio of NMDAR expression. ^#^*p* < 0.05, one-way ANOVA with post hoc Dunnett’s Multiple Comparison Test. ^δ^*p* < 0.05, unpaired student t-test, compared to the same day WT control; *n* is the number of cells recorded from. n.d. indicates not determined due to low current amplitude. n.a. indicates data not available. Part of the data from ^a^Xu et al. [43]; ^b^Lewis et al. [70]; ^c^Fernández-Marmiesse et al. [61]; ^d^Amador et al. [48] and ^e^Platzer et al. [66] are included for comparison

Similar to what was observed for glutamate potency, virtually all variants (40/48) in the M3 transmembrane helix increased the glycine potency (Table 2), including GluN1-A652T which increased glycine potency when expressed only with GluN2B. Only 2/48 variants decreased glycine potency (increased EC_50_ values) by < twofold, 4/48 variants did not detectably alter glycine EC_50_, and 2/48 variants had responses too small to measure. NMDARs containing variants located in M3 transmembrane helix exhibited up to a 75-fold increase in glycine potency; the following variants increased glycine potency by greater than tenfold: GluN1-Y647C, GluN1-Y647S, GluN1-N650I, GluN1-A653G, GluN1-A653T, GluN1-L655Q, GluN2A-A643D, GluN2A-S644G, GluN2A-T646A, GluN2A-T646R, GluN2A-L649V, GluN2A-M653I, GluN2A-M653V, GluN2A-I654T, and GluN2B-Y646C (Table 2). These results suggest that NMDARs that contain M3 variant subunits can be activated with lower concentrations of agonists, and are consistent with the idea that the structural requirements for gating are precise, and selected to reduce ability of low concentrations of glutamate present in the extracellular space (< 80 nM; [22–24]) to open the pore, as most departures from the naturally occurring amino acid within the transmembrane M3 helix enhance the ability of low concentrations of glutamate and glycine to open the channel. While it is possible that the absence of patient-derived variants that decrease agonist potency may reflect detrimental consequences that are incompatible with life, multiple examples of patient-derived variants that reduce glutamate potency are known (e.g., Ref [10]).

### Variants in the M3 transmembrane helix alter sensitivity to endogenous inhibitors

One of the most important features of NMDA receptors is the negative regulation by endogenous extracellular inhibitors such as extracellular protons and Mg^2+^ [1]. NMDARs can be inhibited at low extracellular pH with an IC_50_ value near physiological pH, suggesting they are normally under tonic proton inhibition [25–27]. A central feature of NMDAR function is its voltage-dependent block by extracellular Mg^2+^ (e.g., [28]). We first evaluated the effect of extracellular Mg^2+^ by recording the current responses evoked by 100 μM glutamate and 100 μM glycine with different concentrations of extracellular Mg^2+^ at a holding potential of -60 mV. The data showed that 27 variant NMDARs had a decreased sensitivity to Mg^2+^ (i.e., increased IC_50_ values), compared to only 5 that showed increased sensitivity (decreased IC_50_ values) to extracellular Mg^2+^ (Table 2). The variants GluN1-A637V, GluN1-G638V, GluN1-M641I, GluN2A-S632F, GluN2A-L642R, GluN2A-T646R, GluN2A-F652V, GluN2B-A636V showed particularly strong increases in IC_50_ values (i.e., decreases in potency) greater than 4-fold for Mg^2+^ block at –60 mV, a holding potential selected for proximity to the membrane potential of principal cells (Fig. 2K–O, Table 2). All of these residues with the exception of GluN2A-F652V lie deep in the pore, close to the apex of the reentrant M2 loop, and are well positioned to perturb the M2 residues and alter electrostatic potential on the protein surface (Fig. 1C), which is an important structural determinant of Mg^2+^ block [29–32]. The variants GluN1-A637S, GluN1-G638A, GluN1-M641V, GluN1-V644M, GluN1-A645S, GluN1-N650K, GluN2A-A635T, GluN2A-V639I, GluN2A-L642M, GluN2A-A643D, GluN2A-S644G, GluN2A-T646A, GluN2A-L649V, GluN2A-A650S, GluN2A-M653V, GluN2A-I654T, GluN2B-Y646C, and GluN2B-A652G all showed modest increases in IC_50_ values (i.e., decreases in potency) for Mg^2+^ block at -60 mV (Fig. 2K–O, Table 2). The reduction in sensitivity to Mg^2+^ block will increase current flow at resting membrane potentials and further increase receptor function. Only four variants (GluN1-Y647C, GluN1-N650I, GluN2A-M653I, GluN2B-A639V**)** had decreased IC_50_ values (i.e., increases in potency) for Mg^2+^ block at – 60 mV.

We also evaluated proton sensitivity by comparing the current amplitude recorded from oocytes at pH 6.8 and pH 7.6 at a holding potential of – 40 mV. The majority of the variant NMDARs decreased proton sensitivity because these variants passed more current at pH 6.8 relative to pH 7.6 than WT NMDARs. GluN1 variants with reduced proton sensitivity included GluN1-G638A, GluN1-G638V, GluN1-M641L, GluN1-M641V, GluN1-I643V, GluN1-V644M, GluN1-A645S, GluN1-A653T co-expressed with GluN2A and GluN1-Y647C, GluN1-L655Q co-expressed with GluN2B. In addition, GluN2A-A635T, GluN2A-V639I, GluN2A-L642M, GluN2A-L642R, GluN2A-S644G, GluN2A-T646A, GluN2A-T646R, GluN2A-L649V, GluN2A-A650S, GluN2A-I654T, GluN2B-A636V, GluN2B-A639V, GluN2B-I641T, GluN2B-A652G, GluN2B-A652P and GluN2B-I655F decreased proton sensitivity. These data suggest a reduced sensitivity of gating to inhibition by a physiological concentration of protons at pH 7.4 (which corresponds to ~ 50 nM protons, Fig. 2P, Table 2). A small subset of variants showed increased pH-sensitivity, which was manifest as a lower current response at pH 6.8 relative to pH 7.6 than was observed for WT NMDARs. These variants included GluN1-I642L, GluN1-I642T, GluN1-Y647C, GluN1-Y647S, GluN1-A652T, GluN2A-N648S, GluN2A-F652V, GluN2A-M653I, and GluN2A-M653V (Fig. 2P, Table 2). This is consistent with the idea that most departures from the naturally occurring residues increases channel activation, in this case by reducing tonic proton inhibition present at physiological pH.

### Variants in the M3 transmembrane helix alter divalent ion permeability

An important feature of NMDAR function is its permeability to the divalent cation Ca^2+^, which couples synaptic release of glutamate and depolarization-mediated reduction in Mg^2+^ block to engagement of second messenger systems, post-translational modifications of important proteins, and gene transcription [1]. Variant-induced changes in Ca^2+^ permeability could influence neuronal development, plasticity, and circuit function, and thus could contribute to the clinical phenotype (e.g., [32]). We, therefore, utilized a screen for potential changes to divalent permeability of NMDARs harboring M3 variants expressed in *Xenopus* oocytes. We evaluated the current–voltage relationship and the reversal potential in solutions that contained either 90 mM Na^+^ or 60 mM Ba^2+^ as a charge carrier for 20 representative variants (*see* Methods, [33]) to determine whether there might be systematic changes in Ca^2+^ permeability with variants in M3. We also measured the ratio of current recorded at -75 mV to + 30 mV for solutions containing either Na^+^ or Ba^2+^. A leftward (negative) Ba^2+^-induced shift in the variant NMDAR reversal potential relative to the reversal potential shift observed for WT NMDARs and a reduced current in Ba^2+^ with respect to Na^+^ at -75 mV for variant compared to WT are both consistent with reduced Ba^2+^ permeability. Both a rightward (positive) shift in the variant reversal potential in Ba^2+^ compared to WT NMDARs and an increase in current observed for variant compared to WT NMDAR in Ba^2+^ vs. Na^+^ at -75 mV are consistent with an increased Ba^2+^ permeability. No detectable shift in the reversal potential or the relative current at -75 mV in Ba^2+^ compared to Na^+^ would suggest minimal changes in Ba^2+^ permeability. We identified three variants that showed a negative reversal potential shift (GluN2A-T646A, GluN2A-T646R, GluN2A-N648S) compared to WT receptors, consistent with reduced Ba^2+^ permeability (*see* Methods, Supplemental Fig. S1, Supplemental Table S2). We note that the variant GluN2A-T646R, which places a positive charge in the permeation pathway, produces almost a complete loss of Ba^2+^-mediated inward current. We also identified five variants that showed a positive Ba^2+^-induced reversal potential shift (GluN1-A645S, GluN1-A653G, GluN1-A653T, GluN2A-V639I, GluN2A-I654T) compared to WT receptors, consistent with increased Ba^2+^ permeability (*see* Methods, Supplemental Fig. S1, Supplemental Table S2). These reversal potential changes were accompanied by the predicted changes in relative current for Ba^2+^ to Na^+^ at − 75 mV. All other variants showed less than a 3.4 mV difference in the Ba^2+^-induced reversal potential shift compared to WT (∆∆V_REV_), which we considered below our threshold for detection (*see* Methods). These data indicate that a subset of variants at specific M3 positions (GluN1-645,653 and GluN2A-639,646,648,654) can change Ba^2+^ permeability, which we suggest is predictive of changes in Ca^2+^ permeability. These results suggest more detailed and involved experiments to quantitatively assess the magnitude of these potential changes for the relative permeability of Ca^2+^ to Na^+^ [34] for a subset of M3 variants could be informative.

### Variants in the M3 transmembrane helix alter receptor surface trafficking

Residues in the M3 domain are known to be important regulators of ER retention and surface delivery of NMDA receptors [35, 36]. To investigate if the M3 variants studied here can influence total NMDAR expression or surface expression, we measured the cell surface protein level and total protein level using a reporter assay in which beta-lactamase was fused to the extracellular ATD of WT GluN1 (β-lac-GluN1), WT GluN2A (β-lac-GluN2A), WT GluN2B (β-lac-GluN2B) or the ATD of M3 variant subunit cDNAs. The beta-lactamase cleavage of a cell-impermeable chromogenic substrate in the extracellular solution makes it possible to determine total and surface receptor expression by photometric measurement. ([10, 37]; *see* Methods). The data showed that NMDARs with a M3 variant subunit can reduce surface expression compared to WT, including GluN1-G638V, GluN1-M641I, GluN1-I642L, GluN1-Y647C, GluN1-Y647S, GluN1-N650K, GluN1-A653G, GluN1-L655Q, GluN2A-L642M, GluN2A-L642R, GluN2A-A643D, GluN2A-S644G, GluN2A-T646A, GluN2A-N648S, GluN2A-L649V, GluN2A-A650S, GluN2A-M653I, GluN2A-M653V, GluN2A-I654T, GluN2B-A636P, GluN2B-A636V, GluN2B-A639V, GluN2B-I641T, GluN2B-Y646C, and GluN2B-I655F. Only one variant exhibited over 50% increased surface expression compared to WT controls in this assay, GluN2B-A652G (154 ± 30% of WT, *p* < 0.05) (Table 2). In addition, several variants may alter total NMDAR expression in transfected HEK cells (Supplemental Table S3). These data illustrate the complex nature of variant effects, whereby some variants that alter functional properties in a manner that increases NMDAR currents will at the same time hinder trafficking to the surface, which may appear to reduce current. These data also support previous suggestion that the M3 transmembrane helix controls surface expression [35, 36], and emphasize the need for a comprehensive approach to evaluate all aspects of variant function and trafficking.

### Variants in the M3 transmembrane helix alter response time course

**Fig. 3:**
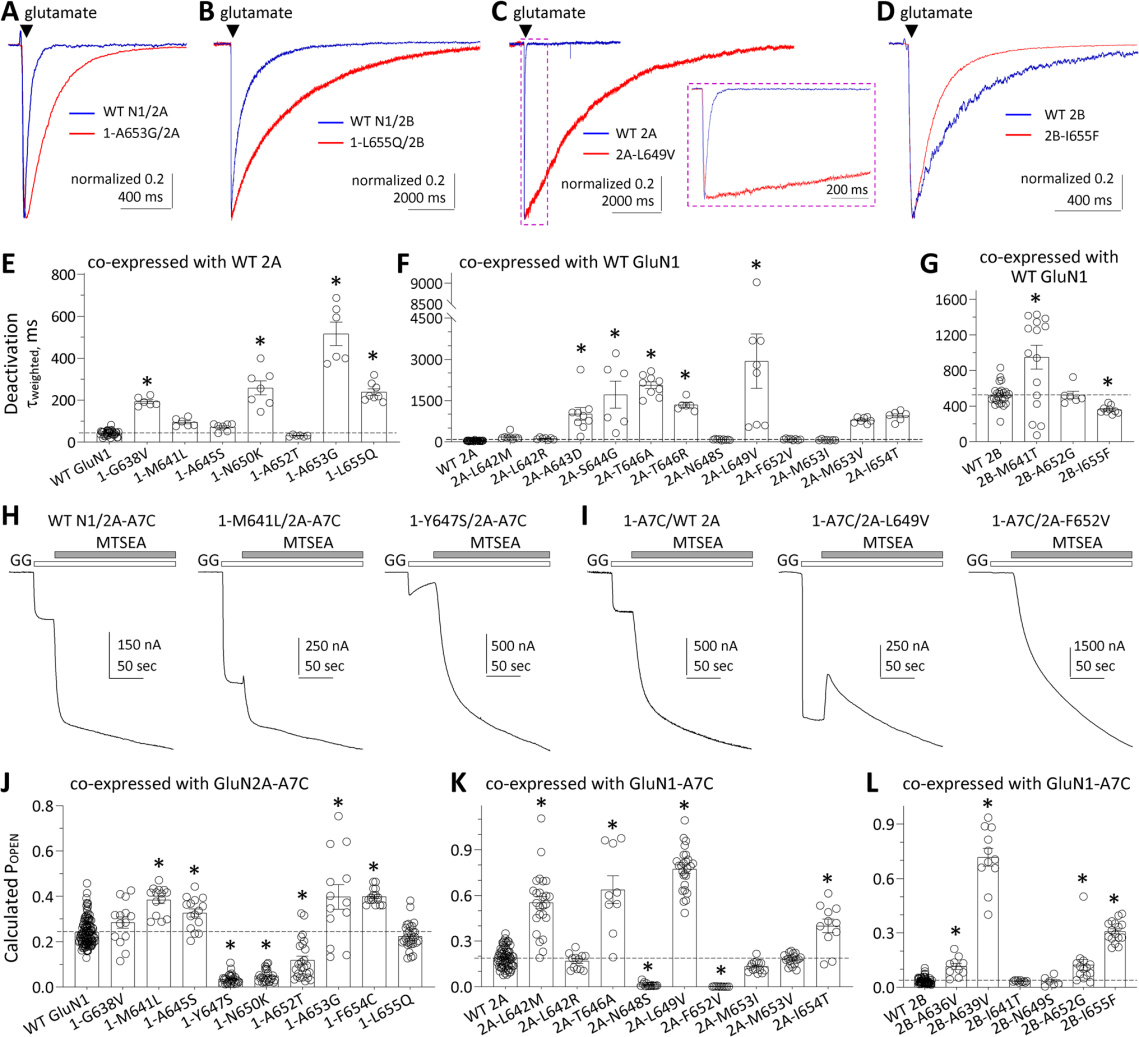
Variants in the M3 transmembrane helix change NMDAR biophysical properties. **A***-***D** Representative whole cell current responses recorded under voltage clamp illustrate the deactivation time course for GluN1/GluN2A, GluN1/GluN2B, GluN1-A653G/GluN2A, GluN1-L655Q/GluN2B, GluN1/GluN2A-L649V, GluN1/GluN2B-I655F NMDARs in response to brief 2–6 ms application and rapid removal of 1 mM glutamate with 100 µM glycine present in all solutions. Variant responses were normalized to the peak amplitude of wild type NMDAR responses. Each variant showed a prolonged deactivation time course compared to WT NMDARs. **E*–*G** Summary of deactivation time course weighted tau for NMDAR variants. **H***, ***I** Representative two electrode voltage clamp current recordings from *Xenopus* oocytes expressing GluN1/GluN2A wild type or variant NMDAR subunits (as indicated) showing current responses evoked by the application of 100 µM glutamate and 100 µM glycine followed by co-application of maximally effective glutamate and glycine plus 0.2 mM MTSEA (*see* Methods). N1-A7C indicates GluN1-A652C and 2A-A7C indicates GluN2A-A650C, which are covalently modified by MTSEA to lock the channel open. **J*–*L** Summary of calculated channel open probability (P_OPEN_) evaluated by the degree of MTSEA potentiation at a holding potential of − 40 mV for GluN1 (**J**), GluN2A (**K**) and GluN2B variants (**L**) (*see* Methods). Data are shown as mean ± SEM

To explore the effects of M3 variants on the deactivation response time course following rapid removal of glutamate from NMDARs, whole-cell patch clamp recordings were conducted on HEK cells transfected with variant GluN1 co-expressed with WT GluN2A, or WT GluN1 co-expressed with variant GluN2. Most of the human M3 variants derived from patients described here altered the deactivation time course compared to WT GluN1/GluN2A (44–51 ms) or WT GluN1/GluN2B (mean 524–663 ms; Fig. 3A–G, Table 3, Supplemental Table S4, S5). This is consistent with the increased agonist potency that often reflects a slower overall rate of agonist unbinding [38, 39]. The dissociation of agonist will often be a rate limiting step during the deactivation time course, although entry into a desensitized state can produce an additional slow component of the deactivation [38]. We observed three variants with strong desensitization in response to prolonged (e.g., 1.5 sec) application of agonists (GluN1-M641L, GluN1-M641V, GluN2B-I641T; *see* Supplemental Fig. S2, Supplemental Table S4). The magnitude of the desensitization of GluN1/GluN2B-I641T varied from cell to cell (Supplemental Fig. S2); the basis for the variability is unclear and may reflect sensitivity of this variant to post-translational modification or other intracellular processes that vary between individual cells.

**Table 3:** Summary of variant NMDAR biophysical properties

	P_OPEN_, MTSEA	Tau_weighted_ (ms)	Amplitude (peak, pA/pF)	Synaptic charge transfer^#^	Non-synaptic charge transfer^#^
WT GluN1/2A	0.24 ± 0.005 (122)	44 ± 2.2 (37)	153 ± 20 (36)	1.0	1.0
1-A637S/2A	0.37 ± 0.05 (16)*	55 ± 4.1 (7)	169 ± 43 (7)	3.4	2.3
1-A637V/2A	0.12 ± 0.01 (15)*	67 ± 8.8 (5)	194 ± 49 (5)	13	15
1-G638A/2A	0.34 ± 0.013 (14)*	88 ± 13 (5)	147 ± 46 (5)	3.4	5.1
1-G638V/2A	0.28 ± 0.03 (14)	194 ± 8.7 (6)	117 ± 37 (6)	39	26
1-M641I/2A^a^	0.095 ± 0.006 (16)*	65 ± 6.7 (6)	73 ± 21 (6)	3.6	2.2
1-M641L/2A	0.38 ± 0.02 (14)*	95 ± 7.8 (6)	139 ± 13 (6)	4.0	11
1-M641V/2A	0.14 ± 0.008 (13)*	49 ± 4.3 (5)	139 ± 24 (5)	1.8	3.5
1-I642L/2A	0.046 ± 0.005 (16)*	23 ± 3.8 (5)	191 ± 50 (5)	0.11	0.075
1-I642T/2A	0.055 ± 0.001 (15)*	27 ± 3.9 (6)	50 ± 22 (5)	0.26	0.17
1-I643V/2A	0.38 ± 0.017 (15)*	163 ± 84 (5)	66 ± 40 (5)	5.7	5.9
1-V644M/2A	0.21 ± 0.009 (20)	1118 ± 335 (5)*	53 ± 22 (5)	111	118
1-A645S/2A	0.34 ± 0.02 (14)*	72 ± 6.6 (7)	234 ± 29 (6)	4.3	3.2
1-Y647C/2A	0.20 ± 0.01 (15)	n.d	0.08 ± 0.02 (6)	–	5.3
1-Y647S/2A	0.027 ± 0.004 (22)*	n.a	n.a	–	1.2
1-N650I/2A	0.14 ± 0.004 (8)*	1753 ± 81 (5)*	37 ± 8.4 (5)	20	33
1-N650K/2A	0.050 ± 0.005 (26)*	259 ± 33 (7)	31 ± 9.5 (7)	0.10	0.28
1-A652T/2A	0.12 ± 0.02 (25)*	31 ± 1.7 (6)	130 ± 56 (6)	0.35	0.52
1-A653G/2A	0.40 ± 0.05 (13)*	516 ± 56 (7)	88 ± 22 (6)	0.80	2.4
1-A653T/2A	0.45 ± 0.01 (16)*	1034 ± 276 (5)*	86 ± 16 (5)	58	114
1-F654C/2A	0.40 ± 0.002 (16)*	351 ± 63 (5)	90 ± 21 (5)	16	53
1-L655Q/2A	0.23 ± 0.007 (31)	239 ± 16 (8)	113 ± 26 (8)	5.3	15
WT GluN1/2A	0.19 ± 0.007 (84)	44 ± 2.2 (37)	153 ± 20 (36)	1.0	1.0
2A-S632F	0.065 ± 0.003 (10)*	46 ± 4.5 (5)	95 ± 34 (5)	1.3	1.4
2A-A635T	0.70 ± 0.03 (10)*	247 ± 43 (5)	59 ± 37 (5)	43	46
2A-V639I	0.99 ± 0.02 (14)*	891 ± 363 (6)*	43 ± 19 (6)	137	79
2A-L642M	0.55 ± 0.04 (25)*	187 ± 38 (8)	79 ± 27 (8)	6.5	19
2A-L642R	0.17 ± 0.01 (12)	121 ± 14 (7)	40 ± 12 (7)	11	21
2A-A643D^b^	0.39 ± 0.02 (20)*	1015 ± 214 (9)*	46 ± 20 (9)	56	28
2A-S644G ^c^	1.0 ± 0.02 (19)*	1718 ± 492 (6)*	87 ± 31 (6)	333	295
2A-T646A	0.64 ± 0.09 (9)*	2059 ± 124 (9)*	72 ± 6.2 (9)	8.2	6.7
2A-T646R	1.0 ± 0.17 (11)*	1341 ± 84 (6)*	19 ± 3.5 (6)	4772	5502
2A-N648S	0.013 ± 0.003 (15)*	88 ± 6.7 (9)	32 ± 4.4 (9)	0.12	0.31
2A-L649V	0.77 ± 0.03 (26)*	2934 ± 990 (8)*	5.5 ± 1.4 (8)	329	304
2A-A650S	0.54 ± 0.03 (17)*	168 ± 44 (5)	97 ± 87 (5)	8.2	4
2A-F652V	0.0018 ± 0.0002 (11)*	97 ± 8.7 (8)	21 ± 17 (8)	0.13	0.31
2A-M653I	0.13 ± 0.01 (14)	75 ± 8.9 (7)	105 ± 25 (7)	0.21	4.3
2A-M653V	0.18 ± 0.09 (18)	795 ± 40 (7)	28 ± 7.6 (7)	26	74
2A-I654T	0.40 ± 0.04 (11)*	948 ± 74 (7)*	24 ± 9.9 (7)	11	31
WT GluN1/2B	0.033 ± 0.002 (84)	524 ± 23 (26)	55 ± 9.7 (26)	1.0	1.0
2B-A636V	0.14 ± 0.02 (16)*	n.d	0.11 ± 0.05 (3)	-	23
2B-A639V	0.72 ± 0.05 (11)*	7954 ± 1726 (5)*	7.2 ± 2.0 (5)	7.9	6.2
2B-I641T	0.034 ± 0.002 (10)	950 ± 134 (16)	33 ± 3.7 (16)	0.55	1.0
2B-Y646C	0.034 ± 0.003 (16)	n.d	0.12 ± 0.04 (7)	–	6.1
2B-N649S	0.017 ± 0.004 (22)	n.d	0.27 ± 0.08 (5)	–	8.2
2B-N649T	0.016 ± 0.005 (20)	n.d	0.47 ± 0.15 (6)	–	8.3
2B-A652G	0.12 ± 0.03 (14)*	525 ± 43 (6)	72 ± 17 (6)	6.3	12
2B-A652P	0.0067 ± 0.0003 (20)	104 ± 11 (5)	56 ± 26 (5)	0.012	0.020
2B-I655F^d^	0.31 ± 0.01 (16)*	361 ± 15 (9)*	93 ± 25 (9)	1.5	2.0

Data were expressed as Mean ± SEM (*n*); n.d. indicates not determined due to low current amplitude

^*^p < 0.05 one-way ANOVA, with Dunnett’s multiple comparisons test, corrected for family wise error by Holm-Bonferroni. ^**#**^The values were calculated by Eqs. 1, 6, 7 and indicate the fold difference in estimated relative synaptic and non-synaptic function for the variants compared to the WT receptors. Part of data are from ^a^[43], ^b^[61], ^c^[48]

**Fig. 4:**
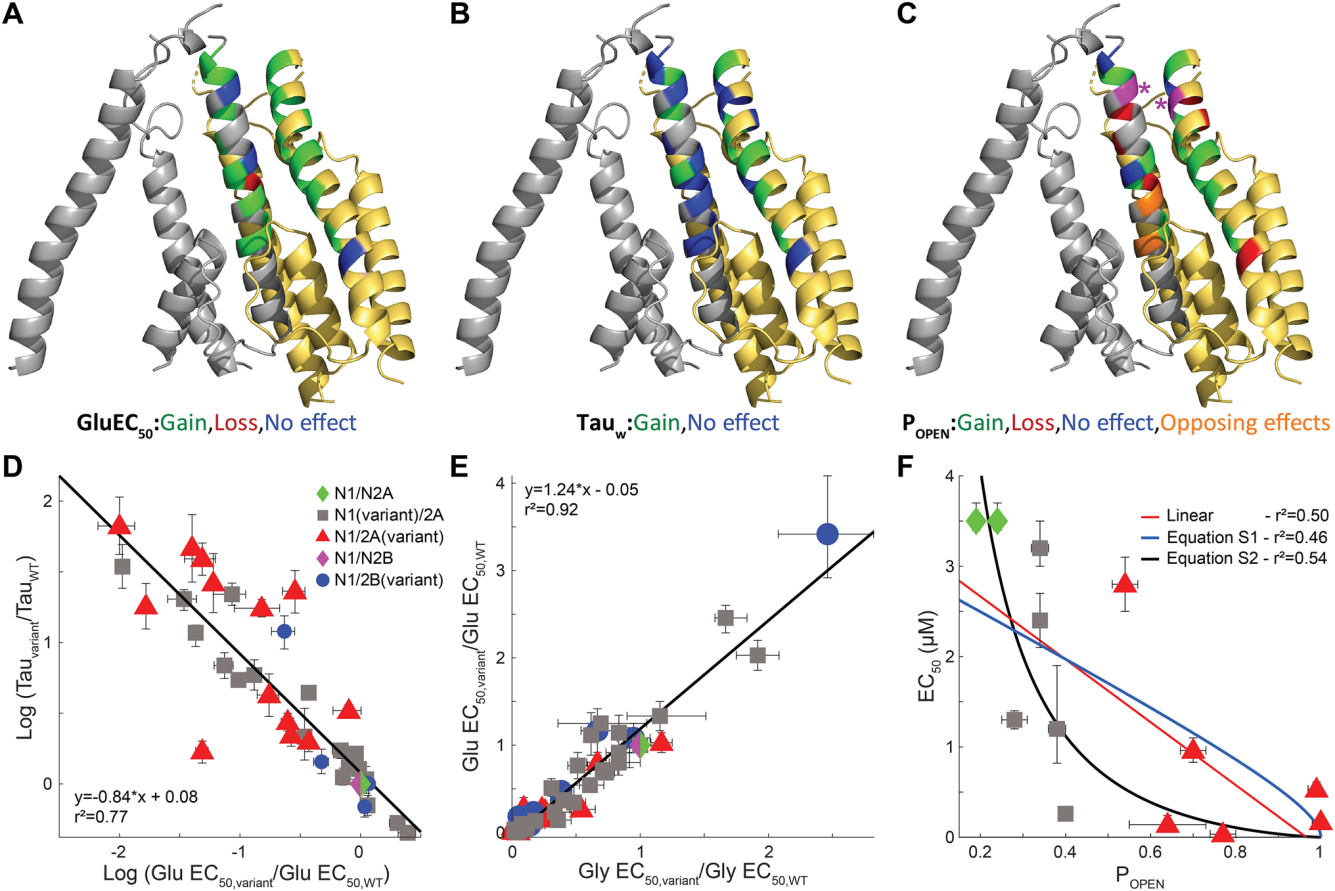
Effects of variants on tau deactivation, glutamate EC_50_, glycine EC_50_, and open probability (P_OPEN_). **A** Measured EC_50_ values for GluN1 and GluN2A variants that were significantly different than WT controls are mapped onto a GluN1/GluN2A homology model of two subunits of the transmembrane pore (GluN1-gray, GluN2 yellow) based on the non-active GluN1/GluN2B structure from Chou et al. [20]. Variants that increase open probability are green, variants that decrease open probability are red, those without an effect are blue, and positions that have multiple variants at the same residue with opposing significantly different effects are orange. Residues that had multiple tested variants which either produced no significant alterations or significant effects in one direction were colored according to observed significant effects. **B** Measured deactivation tau weighted values for GluN1 and GluN2A variants that were significantly different than WT controls are colored as in (**A**). **C** Measured open probability values for GluN1 and GluN2A variants that were significantly different than WT controls are colored as in (**A**). The site at which a Cys residue is substituted and modified by MTSEA on GluN1 (grey) and on GluN2A (yellow) are shown in magenta with an asterisk. **D** The logarithm (Log, base ten) of the ratio of variant to WT tau_weighted_ is plotted against the Log of the ratio of variant to WT glutamate EC_50_. The slope was − 0.84, the intercept was − 0.08, and the value for *R*^2^ was 0.77. **E** The Log of the ratio of variant to WT glycine EC_50_ is plotted against the Log of the ratio of variant to WT glutamate EC_50_. The slope was 1.24, the intercept was -0.05, and the value for R^2^ was 0.92. **F** The measured glutamate EC_50_ value is plotted against Po for variants (GluN1 and GluN2A) that increase open probability compared to WT GluN1/GluN2A NMDARs (*see* Supplemental Fig. S3 for the full dataset). Three regressions are shown; linear fit (*red*, *r*^2^ = 0.50), equation S1 (*blue*, *r*^2^ = 0.46, *see* Supplemental Fig. S3), and equation S2 (*black*, *r*^2^ = 0.54, *see* Supplemental Fig. S3). Error bars depict SEM for Tau and E values and 99% confidence intervals for EC_50_ values

We evaluated the relationship between deactivation and potency, restricting our analysis to EC_50_ values greater than 30 nM, since values lower than this can be impacted by contaminant agonists, which can be present at concentrations of 10’s of nanomolar. Figure 4A shows the position within the M3 transmembrane helix of variants that alter EC_50_. Figure 4B shows the position within the M3 transmembrane helix of variants that alter tau_weighted_ describing the deactivation of the time course. Figure 4D compares the relationship between the experimentally determined EC_50_ values and tau_weighted_. There was a significant correlation between these two parameters when expressed as the Log of the fold changes. The slope was − 0.84 and the intercept was − 0.08, suggesting an expected inverse relationship between EC_50_ and the time course of deactivation. The relationship between EC_50_ and tau_weighted_ appears similar for GluN2A and GluN2B variants over this range of EC_50_ values. Actions on desensitization could influence the weighted time constant for deactivation by altering the rates or probability that a given receptor will enter a long-lived desensitized state from which it must return before unbinding, although we expect that relationship to be more complex. For GluN1 variants, increases in maximal open probability may reduce glutamate EC_50_ (Fig. 4C,F, *see* below) even though GluN1 binds glycine not glutamate. It is known that there is allosteric coupling between glutamate and glycine affinity [40]. Enhanced glutamate potency in GluN1 variants or GluN2 variants could also reflect enhanced gating, which would lead to similar shifts produced by variants in glutamate and glycine potency and was observed in this M3 variant dataset (Fig. 4E).

### Variants in the M3 transmembrane helix alter channel maximal open probability

To evaluate the effects of M3 variants on the maximal open probability for an agonist-bound NMDAR, we measured the MTSEA (2-aminoethyl methanethiosulfonate hydrobromide)-induced potentiation on NMDARs that contained a cysteine mutation introduced into the SYTANLAAF region of GluN1 (GluN1-A652C), GluN2A (GluN2A-A650C) or GluN2B (GluN2B-A651C; [18, 41]). We recorded current responses by TEVC at a holding potential of − 40 mV to 100 μM glutamate and 100 μM glycine, followed by co-application of glutamate and glycine with 200 μM MTSEA, which covalently modified the introduced Cys residue to lock the receptor into the open conformation. Maximal open probability can be calculated as the reciprocal of the degree of potentiation, corrected for a change in single channel conductance. These data suggest that multiple M3-variant-containing NMDARs have significantly higher calculated open probability compared to WT (Fig. 3H–L, Table 3). Notably, GluN2A-T646R showed no MTSEA potentiation (current responses with MTSEA were smaller than the control response), indicating that this variant may interfere with the ability of MTSEA to covalently label and lock the NMDA receptors into the open state. Although most variants increased maximal open probability, there were a few variants (GluN1 Tyr647, Asn650, Ala652 and GluN2A Asn648, Phe652) that decreased open probability, that were close to the residues that were converted to a cysteine (GluN1-A652C, GluN2A-A650C) that is covalently modified by MTSEA to lock the channel open. It is not clear if the reduced open probability in this assay reflects a confounding effect for these variants on the assay readout due to their proximity to the modified residue, or if these residues do indeed lower open probability. Figure 4C illustrates how the results from this assay map onto the pore-lining residues.

There is a well understood relationship between EC_50_ and open probability for all receptors for which agonist binding and channel opening are separated as independent steps [42]. We investigated this relationship by plotting the EC_50_ for variants versus the maximal open probability (Supplemental Fig. S3A). The relationship between open probability and EC_50_ is less clear for maximal open probability values lower than 0.1 [42], and strong reductions to values below 0.1 often reflect processes like desensitization that are only indirectly related to channel opening and closing rates. We observed a trend towards lower glutamate EC_50_ values at higher open probabilities in the data, as expected (Supplemental Fig. S3A). We analyzed GluN1 and GluN2A variants (expressed as GluN1/GluN2A) that showed higher open probabilities than WT and had similar variant-induced fold shifts in glutamate and glycine EC_50_, reasoning that variants that primarily increase open probability will equally impact glutamate and glycine potency. We excluded variants with a ratio of the fold differences for glutamate and glycine that was greater than the mean ± STD (2.7 fold). The data for variants satisfying this criteria were fitted by a linear regression (*r*^2^ = 0.50). We also considered simple gating models that allowed for the relationship between open probability and EC_50_ to be quantified (*see* Supplemental Fig. S3). These models produced a similar fit to the data (*r*^2^ = 0.46–0.54; Fig. 4E, Supplemental Fig. S3), and suggest that there is an underlying relationship between the effects between M3 gain-of-function variants that primarily increase open probability and measured changes in EC_50_.

### Estimating the impact of M3 transmembrane helix variants on NMDAR function

**Fig. 5:**
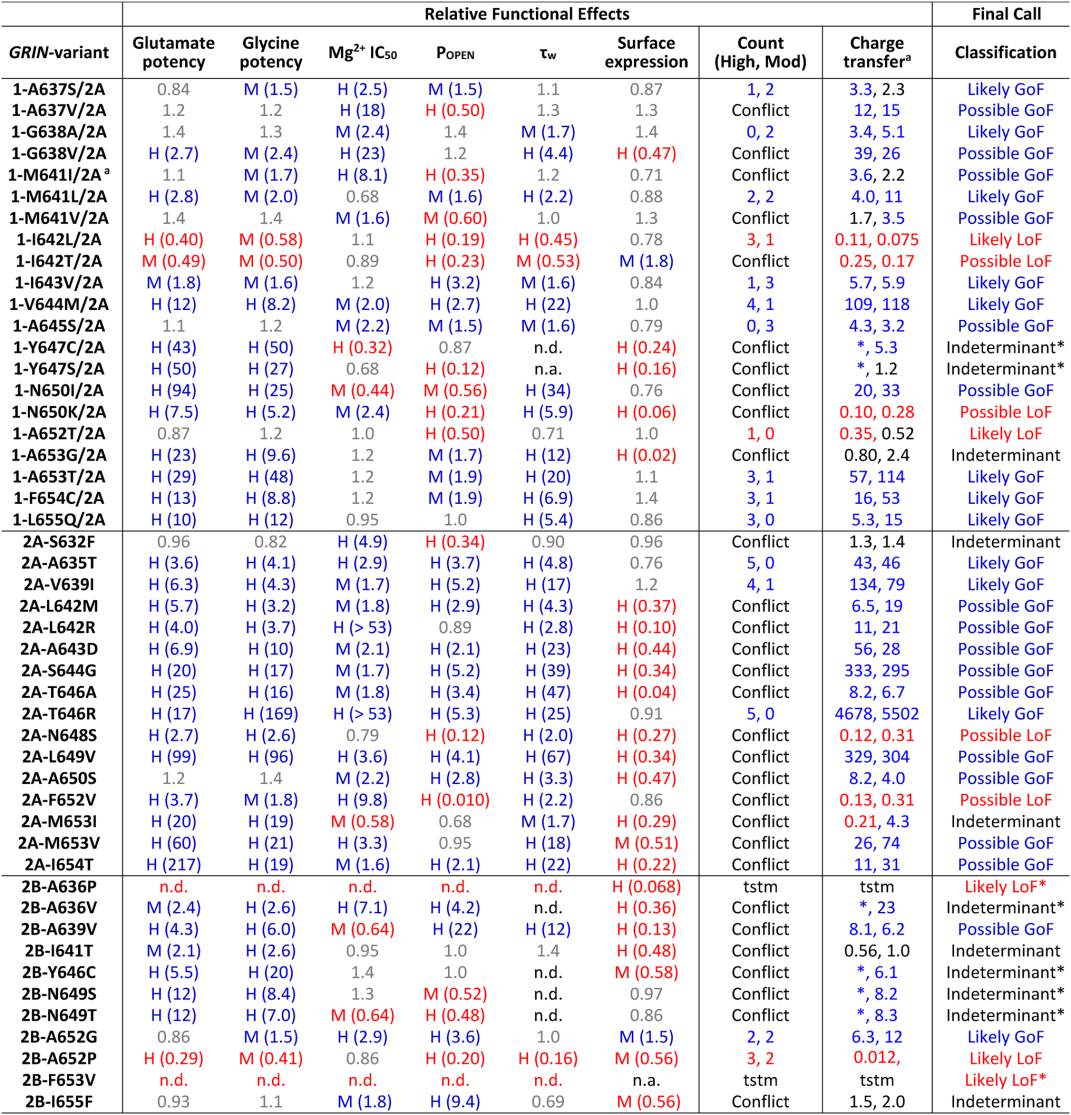
Assessment of M3 variant-mediated changes in parameters supporting GoF and LoF status. Glutamate and glycine potency ratios are given as WT/variant EC_50_ because EC_50_ is reciprocally related to potency. Mg^2+^ IC_50_, open probability (P_OPEN_), weighted tau (τ_w_), and surface expression fold effects are given as variant/WT. Two sets of the pooled τ_w_ data for same day controls were used to obtain WT parameter values for comparison to variants (*see* Supplemental Table S5). A high (H) or moderate (M) confidence (*see* Myers et al. [13]) for the change is indicated; red is LoF and blue is GoF. The number of high (H) and moderate (M) changes are given *Count* when there is no conflict in the direction of change for two parameters. Conflicting and subthreshold variants were re-classified as Possible GoF or Possible LoF if the fold change in the synaptic (left number) or non-synaptic (right number) of relative charge transfer ratio (Variant/WT) was > 2.5-fold or < 0.4-fold. ^a^Calculation of relative for charge transfer (Variant/WT) was from Myers et al., [13]. n.d. indicates response was too small to measure (tstm). ^*^Indicates that experiments to assess tau were run and currents recorded, but they were too small to allow reliable determination of tau and thus we cannot determine whether the variant alters overall function

Genetic variants identified in patients with neurologic disorders usually lead to complex functional changes at the protein, which for NMDARs result in differences in current responses and other properties of the variant receptor, as well as compensatory changes in circuits and systems. To estimate the overall effect of rare variants on NMDAR function and predict their effect on neuronal function (in the absence of compensation), we developed a strategy that considered six measured parameters in addition to an estimation of the relative net change in synaptic and non-synaptic NMDAR charge transfer for variant compared to WT receptors [10, 32]. We completed this analysis for all 48 variants presented here. The majority of the M3 transmembrane helix variants (28/48) shows an enhanced function (Table 3, Fig. 5). The functional enhancement is mainly generated by increased agonist potency, prolonged deactivation time course, and increased maximal open probability, although several variants also showed reduced voltage-dependent Mg^2+^ block. This finding suggests that the arrangement of some amino acids in this region seems critically selected to allow the channel to remain closed so that opening can be  driven by agonist binding. Many amino acid substitutions in M3 appear to perturb the receptor in a manner that reduces the activation energy required for gating and leads to apparent gain-of-function that is manifest as larger charge transfer both for responses to synaptic glutamate profile (27/48 showed greater than 2.5-fold increase [13]) and/or steady-state low glutamate concentration (32/48 showed greater than 2.5-fold increase [13]), as one would expect to find extrasynaptically [22–24]. We found that 9/48 variant appeared to reduce NMDAR function, 3 of which were influenced by strong reduction (e.g.  > 90%) in trafficking to the plasma membrane.

### Rescue pharmacology of variants in M3 transmembrane helix

**Fig. 6:**
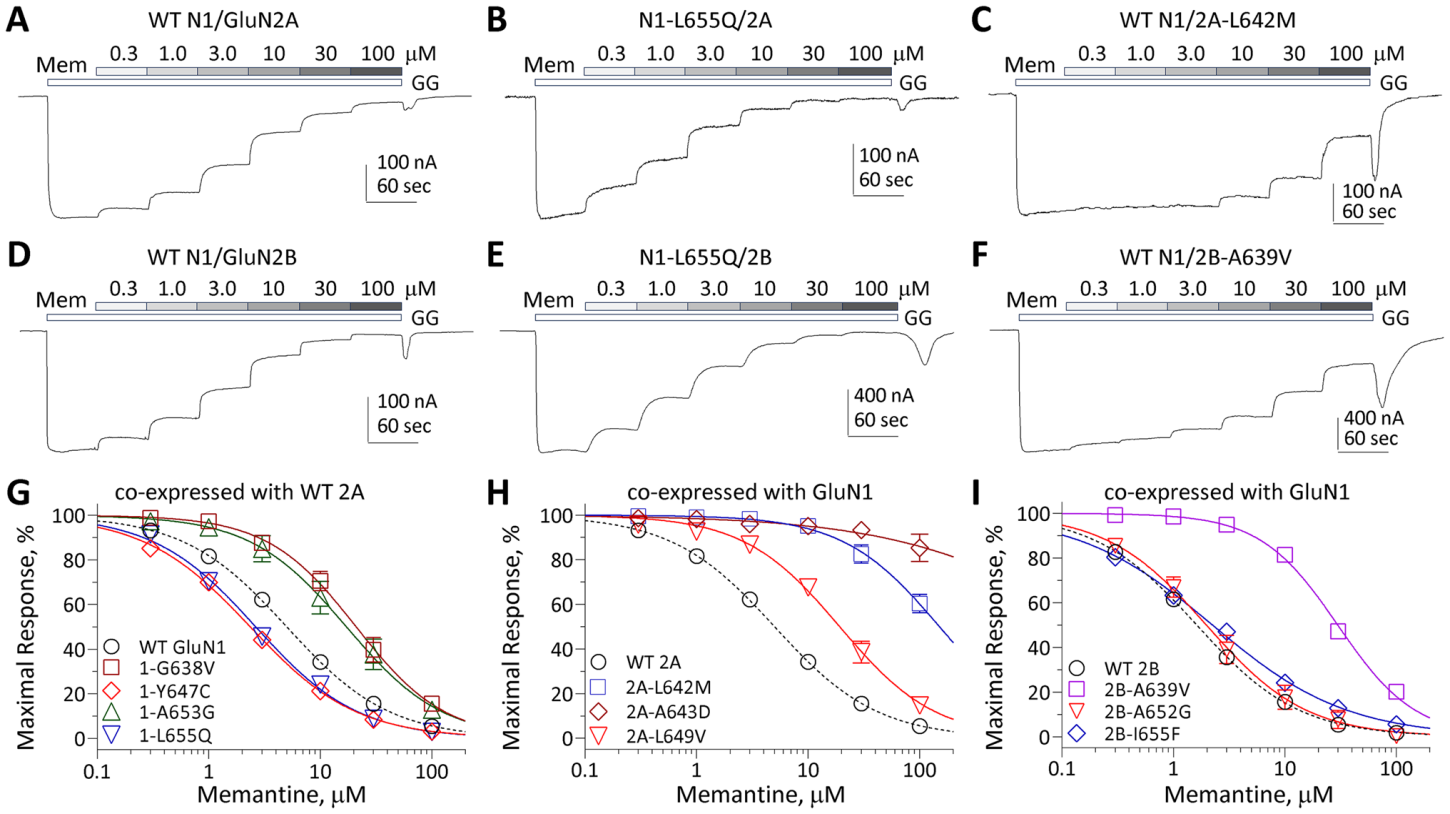
Variants in the M3 transmembrane helix influence sensitivity to FDA-approved NMDAR channel blocker memantine. **A-F** Representative two electrode voltage clamp current recordings from *Xenopus* oocytes expressing GluN1/GluN2A and GluN1/GluN2B WT or variant NMDAR subunits, as indicated. The FDA-approved NMDAR channel blocker memantine concentration–response relationship was determined by co-applying increasing concentrations of memantine (Mem) with maximally effective concentrations of glutamate and glycine (100 µM) at a holding potential of − 40 mV. **G-I** Composite concentration–response curves of memantine were assessed by TEVC recordings of *Xenopus* oocytes for GluN1 (**G**), GluN2A (**H**) and GluN2B (**I**) variants in the presence of maximally effective concentrations of agonists (100 µM glutamate and 100 µM glycine) at a holding potential of − 40 mV. Data are shown as mean ± SEM

**Fig. 7:**
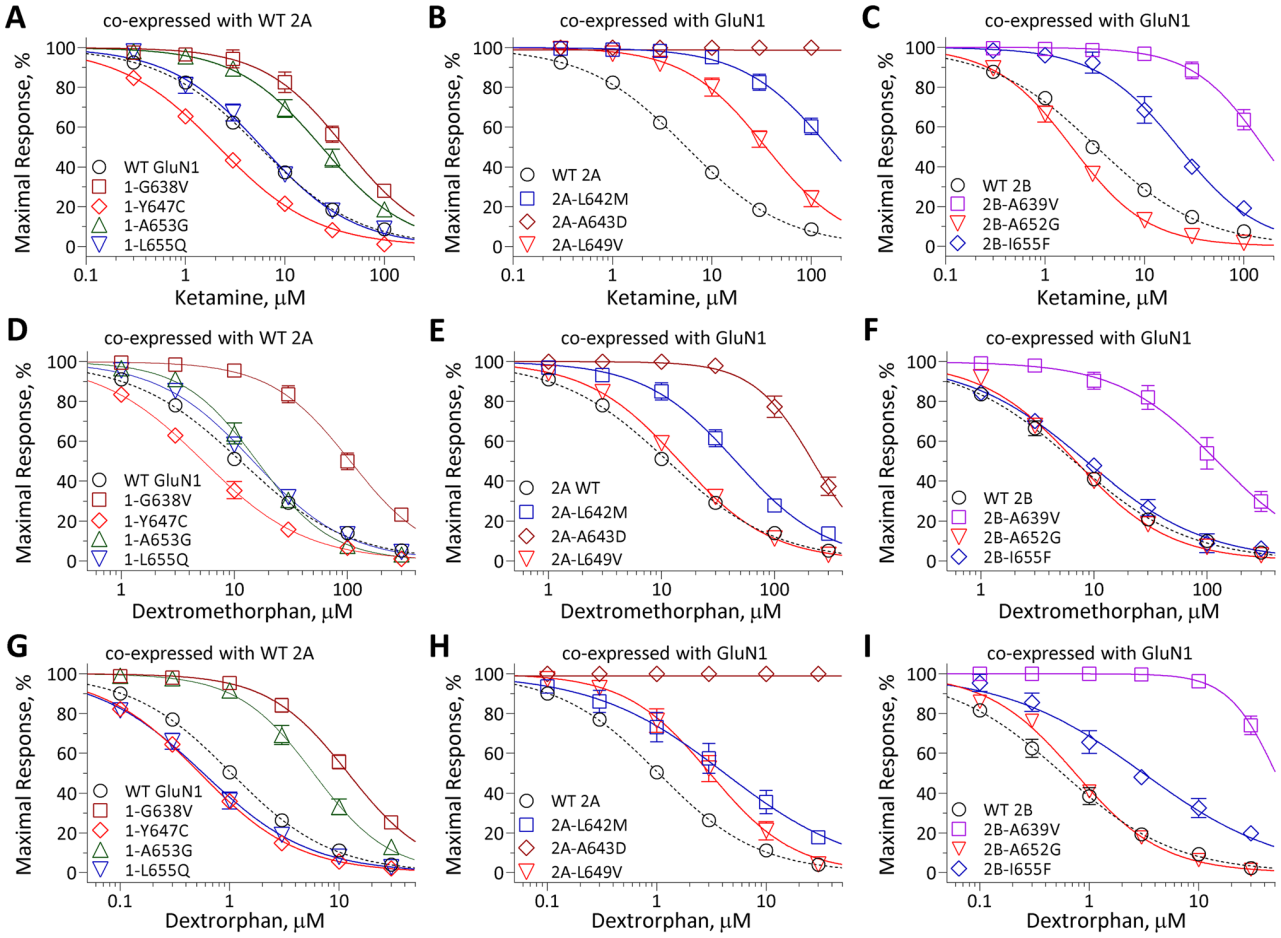
Variants in the M3 transmembrane helix influence sensitivity to FDA-approved NMDAR channel blockers ketamine, dextromethorphan and its metabolite dextrorphan. Composite concentration–response curves of FDA-approved NMDAR channel blockers ketamine (**A–C**), dextromethorphan **(D–F)** and its CYP2D6 metabolite dextrorphan **(G–I)** were assessed by TEVC recordings of *Xenopus* oocytes in the presence of maximally effective concentrations of agonists (100 µM glutamate and 100 µM glycine) at holding potential of – 40 mV. Data are shown as mean ± SEM

The functional data described here suggest that a significant proportion of M3 variants produces a gain-of-function, which means that these variant NMDARs will be more active on the cell surface. That is, the variant NMDARs are more likely to drive larger inward currents and produce greater Ca^2+^ entry into the cell each time they are activated compared to WT. To determine whether FDA-approved NMDAR channel blockers might be useful to potentially mitigate some of the effects of overactive NMDARs, we used TEVC recordings from *Xenopus* oocytes expressing WT and variant NMDARs to determine the IC_50_ values for these compounds (Figs. 6, 7; Table 4). In general, most M3 variant NMDARs showed a decreased sensitivity (increased IC_50_ values) to memantine, ketamine, dextromethorphan, and the metabolite dextrorphan. This was especially evident for GluN2A-L642M, GluN2A-L642R, GluN2A-A643D, GluN2A-T646A and GluN2B-A639V (Figs. 6, 7; Table 4). However, a subset of variants residing near the upper portion of the internal cavity increased blocker potency, raising the possibility that they could be useful for selectively reducing current through variant but not WT receptors (e.g., [43, 44]). This phenomenon is consistent with the effect of variants in this region, as illustrated in structural studies of therapeutically-relevant channel blockers such as memantine and ketamine [45].

**Table 4:** Summary of rescue pharmacology

	Memantine IC_50_, µM (*n*)	Ketamine IC_50_, µM (*n*)	Dextromethorphan IC_50_, µM (*n*)	Dextrorphan IC_50_, µM (*n*)
WT N1/WT 2A	5.1 [4.3, 5.9] (61)	6.3 [4.8, 8.2] (32)	13 [10, 16] (41)	1.1 [0.93, 1.2] (48)
1-G638V/2A	21 [15, 30] (14)*	36 [22, 58] (17)*	105 [79, 141] (16)*	12 [9.2, 15] (19)*
1-M641I/2A^a^	1.3 [0.92, 1.6] (23)*	0.94 [0.86, 1.0] (8)*	0.42 [0.33, 0.52] (9)*	0.080 [0.064, 0.10] (10)*
1-M641L/2A	2.3 [1.7, 3.2] (11)*	2.1 [1.7, 2.6] (8)*	5.5 [4.1, 7.5] (14)*	0.54 [0.43, 0.69] (13)*
1-A645S/2A	36 [26, 49] (13)*	> 100 (14)*	79 [51, 122] (10)*	12 [8.0, 17] (13)*
1-Y647C/2A	2.3 [1.7, 3.2] (11)*	2.1 [1.7, 2.6] (8)*	5.5 [4.1, 7.5] (14)*	0.54 [0.43, 0.69] (13)*
1-Y647S/2A	0.71 [0.57, 0.87] (13)*	n.a	5.2 [4.2, 6.4] (14)*	0.60 [0.41, 0.86] (12)*
1-N650K/2A	1.5 [1.2, 1.9] (22)*	2.4 [1.7, 3.4] (26)*	4.1 [3.2, 5.1] (15)*	0.26 [0.19, 0.36] (12)*
1-A653G/2A	16 [8.6, 29] (12)*	23 [16, 32] (14)*	15 [11, 20] (12)	5.7 [4.3, 7.5] (16)*
1-F654C/2A	8.7 [8.1, 9.3] (11)*	11 [10, 12] (10)*	32 [28, 36] (10)*	2.0 [1.8, 2.1] (14)*
1-L655Q/2A	2.7 [2.1, 3.5] (12)*	6.4 [4.9, 8.2] (12)	15 [12, 17] (13)	0.56 [0.38, 0.84] (14)*
2A-L642M	> 100 (24)*	> 100 (13)*	43 [33, 56] (10)*	3.7 [1.7, 8.1] (8)*
2A-L642R	56 [39, 80] (11)*	> 100 (14)*	45 [30, 68] (15)*	17 [11, 29] (6)*
2A-A643D	> 100 (12)*	> 100 (16)*	210 [163, 270] (15)*	> 30 (4)*
2A-S644G	11 [7.3, 15] (15)*	> 100 (14)*	15 [8.7, 27] (6)	1.4 [0.68, 2.8] (13)
2A-T646A	> 100 (12)*	> 100 (12)*	> 300 (12)*	> 30 (12)*
2A-L649V	19 [15, 25] (7)*	35 [25, 48] (8)*	14 [11, 18] (10)	3.0 [1.9, 4.9] (15)*
2A-A650S	8.5 [7.1, 10] (8)*	78 [45, 137] (7)*	45 [33, 60] (9)*	3.1 [2.4, 4.0] (10)*
2A-M653I	12 [9.2, 15] (14)*	10 [6.0, 17] (12)	> 300 (8)*	17 [11, 26] (9)*
2A-M653V	6.4 [5.2, 7.9] (15)	12 [7.1, 20] (14)	35 [26, 47] (10)*	4.1 [2.9, 5.8] (19)*
2A-I654T	37 [30, 45] (10)*	20 [11, 34] (12)*	> 300 (6)*	> 30 (10)*
WT N1/WT 2B	1.6 [1.3, 2.0] (19)	3.1 [2.2, 4.4] (14)	6.6 [5.1, 8.6] (12)	0.57 [0.38, 0.83] (24)
1-M641I/2B^a^	1.3 [0.92, 1.6] (23)	0.94 [0.86, 1.0] (8)*	0.42 [0.33, 0.52] (9)*	0.080 [0.064, 0.10] (10)*
1-L655Q/2B	1.5 [1.4, 1.8] (6)	n.a	5.9 [4.9, 7.1] (6)	0.25 [0.20, 0.31] (7)*
2B-A639V	29 [25, 34] (14)*	> 100 (16)*	103 [58, 183] (14)*	> 30 (14)*
2B-A652G	2.0 [1.2, 3.5] (6)	1.8 [1.4, 2.2] (8)*	7.0 [5.5, 8.9] (6)	0.75 [0.68, 0.83] (5)
2B-I655F	2.2 [1.8, 2.5] (6)	21 [13, 35] (6)*	9.3 [6.4, 13] (8)	1.9 [1.1, 3.5] (18)*

The concentration–response relationship for FDA-approved NMDAR drugs was determined in the presence of 0.1 mM glutamate and 0.1 mM glycine by TEVC recordings (V_HOLD_: − 40 mV). Data shown are the mean IC_50_ value with 95% confidence intervals determined from the LogIC_50_ values

*Indicates 95% confidence intervals that are non-overlapping with WT GluN1/GluN2A or WT GluN1/GluN2B NMDARs. *n* is the number of oocytes recorded from. n.a. indicates data not available

^a^Data are from Xu et al. [43]

## Discussion

In this study, we describe 48 missense variants in the M3 transmembrane helix encoded by various *GRIN* genes from 56 patients. According to the available clinical data, the most common phenotypes of these patients with M3 transmembrane variants are epilepsy, intellectual disability, and developmental delay. Evaluation of the genetic variation across the functional domains of GluN1 and GluN2 revealed that most residues within the M3 transmembrane helix (except those in GluN2C) are subject to purifying selection [21] (Fig. 1), suggesting that functional variation therein is likely to be detrimental and pathogenic. This analysis strongly suggests that future variants found in the M3 transmembrane helix are likely to be dysfunctional and potentially contribute to the patients’ clinical phenotype.

An important point of consideration is the fact that properties assessed in this study are from NMDARs that contain two copies of the variant subunit, which will be a minority of receptors in patients. Most receptors in patients will contain a single copy of the variant subunit. This raises the question of whether the results obtained from the study of receptors with two copies of the variant subunit are predictive of receptors with a single copy of the variant subunit. A number of studies evaluating NMDARs that contain one or two copies of the variant subunit suggest that the changes in properties found in NMDARs with two copies of the variant subunit are similar to those that occur in receptors with a single copy, although the magnitude of the change is often less. For example, 15 *GRIN* variants including GluN1-G620R [32], GluN2A-P552R [46, 47], GluN2A-L611Q, GluN2A-N614S, GluN2A-N615K [32], GluN2A-S644G [48], GluN2A-D731N [49], GluN2A-L812M [16], GluN2A-M817V [14], GluN2B-E413G [10], and GluN2B-W607C, GluN2B-G611V, GluN2B-N615K, GluN2B-V618G, GluN2B-V620M [32] that have been studied show qualitatively similar changes in diheteomeric receptor properties occur when a single copy of a variant subunit is present compared to when two copies of a variant subunit are present. Consistent with this result, we find similar effects on glutamate and glycine potency for NMDARs with two copies of the GluN2A-L642M variant subunit and NMDARs harboring a single copy of this variant subunit (*see* Supplemental Fig. S4). In addition, GluN1/GluN2A/GluN2B triheteromeric receptors that contained one copy of either GluN2A-E551K, GluN2A-P552R, GluN2A-S644G, GluN2A-L649V, GluN2A-L812M, GluN2B-G543R, GluN2B-A639V, GluN2B-M818T, or GluN2B-A819T [50] show similar changes compared to diheteromeric receptors with two copies of the variant subunit. Some of these published variants (GluN2B-A639V, GluN2A-S644G, GluN2A-L649V) for which properties in receptors with two copies are predictive of effects on receptors with a single copy of the variant subunit [48, 50] were evaluated here in this study, and this previously published work confirms that their properties transfer to NMDARs with a single variant subunit. These data support the idea that the qualitative properties identified in NMDARs with two copies of the variant subunit are predictive of properties with a single copy of the subunit.

The impact of M3 variants on NMDAR function is comprehensive and includes changes in agonist potency, sensitivity to negative allosteric modulators, receptor surface trafficking, deactivation time course, and divalent ion permeability. Combining relative changes for most of these assays, we can predict the potential for gain- or loss-of-function for these variants [10, 32]. Most of the M3 variants evaluated in this study showed an increased potency to glutamate and glycine, suggesting that variants within the M3 domain often make the receptor more effective. For models in which binding is separated from gating steps, the coupling of EC_50_ to efficacy (i.e., open probability here) is implicit in the solution to the system of equations describing the model. For example, Colquhoun [42] shows that increases in efficacy alone without changes in association or dissociation rates decrease agonist EC_50_. This likely reflects a lower activation energy by the M3 variant for progression along steps that lead to pore opening, which is well-known to increase the potency of agonist by shifting the equilibrium toward open states. Although the consequent increased current expected from M3 variation could be homeostatically compensated by overall lower surface expression, we predict that variants within this region found in the future are most likely to be GoF, provided that they reach the neuronal surface. However, we also find some notable exceptions in these variants. For example, at the locus GluN2A-L642, different amino acid substitutions such as methionine and arginine produce a large difference in the sensitivity to Mg^2+^ block. GluN2A-L642M showed no significant difference compared to WT GluN2A, whereas GluN2A-L642R is insensitive to extracellular Mg^2+^, which is likely a consequence of the introduction of a positive charge near the Mg^2+^ binding site. GluN2A-T646A and GluN2A-T646R showed a similar difference, again consistent with the introduction of a charge within the pore that perturbs Mg^2+^ binding. These data suggest that variants within or close to the conserved SYTANLAAF motif whose side chains face the ion channel pore could have differential effects dependent on the characteristics of the side chain.

For patients of drug-resistant epilepsy with *GRIN* gene variants, FDA-approved NMDAR blockers might provide some reduction in excitation and possibly epileptiform activity, with potential utility as antiseizure medications [43, 48, 51, 52]. The evaluation of FDA-approved NMDAR channel blockers suggests that most of the GoF M3 variant NMDARs decreased the sensitivity to ketamine, memantine, dextrorphan and dextromethorphan, suggesting that these drugs are unlikely to be suitable for patients with certain *GRIN* variants, since the drugs do not reach levels in brain necessary to block variant NMDARs. By contrast, some variants near the extracellular facing surface of the internal pocket (Fig. 1B) increase sensitivity to channel blockers (GluN1-Y647C, GluN1-L655Q, GluN2A-N648S, and possibly GluN2B-A652G), and patients with these variants might find that these channel blockers mitigate some of the consequences of the variants. This is consistent with recent structural work that suggests that this region of the intra-pore cavity can interact with channel blockers [45]. However, the shallow current–voltage curves produced by monovalent organic channel blockers, while mitigating excess activation, are unlikely to possess the voltage-dependence of Mg^2+^ which is likely necessary to allow the receptors to act as coincidence detectors, and thus full restoration of synaptic plasticity might not be possible with these drugs [44].

In summary, this study shows for the first time that the most common functional consequences of variation in an intolerant portion of the NMDAR comprising the M3 transmembrane helix, which is known to control channel gating, are enhanced activity categorized as a gain-of-function. These data suggest that future variants in the M3 region of *GRIN1* or any *GRIN2* gene identified in patients will have similar effects and lead to similar clinical phenotypes. It further stresses the significance of the combination of clinical data, genomic sequencing, functional validation and in vitro rescue pharmacology to provide not only a better understanding of pathogenesis but precise strategy of treatment as well. In addition, it provides insight into the requirements for residues at some positions within this gating region, as random variants arising from single nucleotide polymorphisms often increase channel activation. This would be consistent with the need for the pore to remain closed except when coupled to energy produced by agonist binding.

### Supplementary Information

Below is the link to the electronic supplementary material.

Supplementary file1 (XLSX 33 KB)

Supplementary file2 (PDF 760 KB)

## Data Availability

The datasets generated during and/or analyzed during the current study are available from the corresponding author on reasonable request.

## Abbreviations

ABD: Agonist binding domain
ASD: Autism spectrum disorder
CTD: Cytosolic carboxyl terminal domain
DEE: Developmental and epileptic encephalopathy
EOEE: Early-onset epileptic encephalopathy
EPI: Epilepsy/seizures
ADHD: Attention deficit hyperactivity disorder
ID: Intellectual disability
IS: infantile spasms
GoF: Gain-of-function
LD: Language disorder
LoF: Loss-of-function
MD: Movement disorder
NMDAR: N-methyl-d-aspartate receptor
NTD: N-terminal domain, also ATD
TMD: Transmembrane domains (M1, M3, M4, and a reentrant loop M2)
