# Supplementary material for: De novo GRIN variants in M3 helix associated with neurological disorders control channel gating of NMDA receptor
Source: Cell Mol Life Sci. 2024 Mar 28;81(1):153. doi: 10.1007/s00018-023-05069-z (PMC10973091; doi:10.1007/s00018-023-05069-z)
Supplement: Supplementary file 2 — Supplementary file2 (PDF 760 KB) [file 18_2023_5069_MOESM2_ESM.pdf]

Supplemental Information

De novo GRIN variants in M3 helix associated with neurological disorders control channel gating of NMDA receptor

Yuchen Xu[1,20], Rui Song[1,21], Riley E. Perszyk[1], Wenjuan Chen[1,22], Sukhan Kim[1,2], Kristen L. Park[3], James P. Allen[1], Kelsey A. Nocilla[1], Jing Zhang[1], Wenshu XiangWei[1,23], Anel Tankovic[1], Ellington D. McDaniels[1], Rehan Sheikh[1], Ruth K. Mizu[1], Manish M. Karamchandani[1], Chun Hu[1], Hirofumi Kusumoto[1], Joseph Pecha[1], Gerarda Cappuccio[4,24], John Gaitanis[5], Jennifer Sullivan[6], Vandana Shashi[6], Slave Petrovski[7,8], Robin-Tobias Jauss[9], Hyun Kyung Lee[10], Xiuhua Bozarth[11,12], David R. Lynch[13], Ingo Helbig[14], Tyler Mark Pierson[15,16,17], Cornelius F. Boerkoel[10], Scott J. Myers[1,2], Johannes R. Lemke[9,18], Timothy A. Benke[3], Hongjie Yuan[1,2*], Stephen F. Traynelis[1,2,19*]

Tables of Contents

Supplemental Table S1. Clinical features and genetic characteristic of patients with *GRIN* variants located in the M3 domain
Supplemental Table S2. Summary of variant NMDAR barium permeability
Supplemental Table S3. Summary of total variant NMDAR expression
Supplemental Table S4. Summary of variant NMDAR response time course
Supplemental Table S5. WT controls of NMDAR response time course

Supplemental Figure S1. M3 variants alter variant NMDAR barium permeability
Supplemental Figure S2. M3 variants that alter variant NMDAR desensitization
Supplemental Figure S3. Non-linear transformations used to evaluate the correlation between glutamate EC_{50} and maximum open probability (P_{OPEN})
Supplemental Figure S4. Effects of a single copy of GluN2A-L642M on agonist potency

References

Supplemental Table S1: Clinical features and genetic characteristic of patients with GRIN variants located in the M3 domain

See EXCEL file

Supplemental Table S2. Summary of variant NMDAR barium permeability

Variant	Na^+ Vrev(mV)	Ba^{2+} Vrev(mV)	Vrev(Ba^{2+})-Vrev(Na^+) ΔVrev (mV)	Variant-WT ΔΔVrev (mV)	Na^+ I(-75mV)/I(+30mV)	Ba^{2+} I(-75mV)/I(+30mV)	I(-75mV Ba)/ I(-75mV Na)	Mg^{2+} IC_{50} (uM)	N
WT N1/2A	-3.9	-15.5	-11.6	0.0	-2.63	-0.85	0.32	19	16
1-A637V/2A	-4.9	-17.0	-12.2	-0.6	-2.69	-0.72	0.27	347	6
1-G638A/2A	-1.6	-15.9	-14.2	-2.7	-2.43	-0.90	0.37	46	5
1-G638V/2A	-2.5	-15.5	-13.1	-1.5	-2.23	-0.65	0.29	435	5
1-M641I/2A	-3.1	-14.8	-11.7	-0.1	-2.26	-0.50	0.22	161	6
1-M641L/2A	-3.4	-16.0	-12.6	-1.0	-2.52	-0.70	0.28	13	6
1-M641V/2A	-4.2	-16.8	-12.6	-1.0	-2.22	-0.87	0.39	31	4
1-V644M/2A	-0.6	-12.5	-11.9	-0.3	-3.58	-1.47	0.41	38	5
1-A645S/2A	-6.0	-10.7	-4.7	6.9	-1.90	-1.26	0.66	41	6
1-N650I/2A	-2.1	-13.2	-11.1	0.5	-2.73	-0.85	0.31	8.3	5
1-N650K/2A	-3.3	-14.4	-11.2	0.4	-2.28	-0.51	0.22	46	5
1-A652T/2A	-5.3	-16.1	-10.7	0.9	-1.63	-0.59	0.36	19	6
1-A653G/2A	-8.6	-13.6	-5.0	6.6	-1.65	-1.40	0.85	22	6
1-A653T/2A	-5.8	-12.2	-6.4	5.2	-2.15	-1.40	0.65	23	5
1-F654C/2A	-5.4	-14.5	-9.1	2.5	-2.05	-1.09	0.53	22	5
N1/2A-V639I	-8.1	-12.9	-4.9	6.7	-2.11	-1.44	0.68	33	5
N1/2A-T646A	1.6	-19.3	-20.9	-9.3	-3.34	-0.39	0.12	35	6
N1/2A-T646R	-1.9	-53.6	-51.7	-40.1	-1.82	-0.08	0.04	>1000	4
N1/2A-N648S	-2.2	-20.6	-18.4	-6.8	-1.65	-0.44	0.27	15	6
N1/2A-M653I	-3.3	-15.9	-12.7	-1.1	-2.13	-1.35	0.63	11	5
N1/2A-I654T	-6.7	-0.9	5.8	17.4	-1.83	-2.47	1.35	68	5

Receptors showing more than a ±3.4 mV change in the ΔΔVrev are shown in red when the change should decrease Ba^{2+} permeability and in blue when the change should increase Ba^{2+} permeability, which we assume is an estimate fold changes in Ca^{2+} permeability. N is the number of oocytes recorded.

Supplemental Table S3. Summary of variant NMDAR total expression

	Total (/WT ratio)
**WT GluN1/2A**	1.0 (34)
**1-A637S/2A**	0.58 ± 0.25 (3)
**1-A637V/2A**	1.41 ± 0.09 (4) [δ]
**1-G638A/2A**	1.11 ± 0.05 (4)
**1-G638V/2A**	0.36 ± 0.06 (5) [δ]
**1-M641I/2A**	0.87 ± 0.18 (5)
**1-M641L/2A**	0.79 ± 0.03 (4) [δ]
**1-M641V/2A**	1.07 ± 0.13 (6)
**1-I642L/2A**	0.81 ± 0.20 (4) [δ]
**1-I642T/2A**	0.99 ± 0.37 (3)
**1-I643V/2A**	0.95 ± 0.017 (6)
**1-V644M/2A**	1.57 ± 0.13 (4) [δ]
**1-A645S/2A**	0.78 ± 0.19 (5)
**1-Y647C/2A**	1.0 ± 0.16 (4)
**1-Y647S/2A**	0.68 ± 0.16 (4)
**1-N650I/2A**	0.60 ± 0.08 (6) [δ]
**1-N650K/2A**	0.11 ± 0.04 (5) [δ]
**1-A652T/2A**	0.91 ± 0.11 (6)
**1-A653G/2A**	0.066 ± 0.004 (6) [δ]
**1-A653T/2A**	1.26 ± 0.08 (4) [δ]
**1-F654C/2A**	1.31 ± 0.10 (4) [δ]
**1-L655Q/2A**	0.60 ± 0.12 (5) [δ]

	Total (/WT ratio)
**WT GluN1/2A**	1.0 (42)
**2A-S632F**	0.49 ± 0.03 (8) [δ]
**2A-A635T**	0.22 ± 0.05 (10) [δ]
**2A-V639I**	0.39 ± 0.01 (6) [δ]
**2A-L642M**	0.43 ± 0.082 (4) [δ]
**2A-L642R**	0.35 ± 0.12 (4) [δ]
**2A-A643D** [c]	0.44 ± 0.15 (4) [δ]
**2A-S644G** [d]	0.58 ± 0.16 (4) [δ]
**2A-T646A**	0.24 ± 0.05 (4) [δ]
**2A-T646R**	0.63 ± 0.04 (6) [δ]
**2A-N648S**	0.08 ± 0.03 (6) [δ]
**2A-L649V**	0.84 ± 0.36 (4)
**2A-A650S**	0.88 ± 0.24 (5)
**2A-F652V**	1.02 ± 0.10 (4)
**2A-M653I**	0.47 ± 0.10 (4) [δ]
**2A-M653V**	0.29 ± 0.08 (3) [δ]
**2A-I654T**	0.32 ± 0.08 (4) [δ]

	Total (/WT ratio)
**WT GluN1/2B**	1.0 (10)
**1-G638V/2B**	0.85 ± 0.22 (4)
**1-M641I/2B** [a]	2.1 ± 0.23 (6) [δ]
**1-A645S/2B**	1.47 ± 0.23 (6)
**1-Y647C/2B**	1.39 ± 0.14 (4)
**1-N650K/2B**	0.15 ± 0.03 (4) [δ]
**1-A652T/2B**	1.33 ± 0.23 (4)
**1-A653G/2B**	0.05 ± 0.005 (4) [δ]
**1-L655Q/2B**	1.10 ± 0.16 (4)

	Total (/WT ratio)
**WT GluN1/2B**	1.0 (38)
**2B-A636P**	0.48 ± 0.14 (4) [δ]
**2B-A636V**	0.51 ± 0.09 (10) [δ]
**2B-A639V**	0.20 ± 0.09 (4) [δ]
**2B-I641T**	0.84 ± 0.14 (4)
**2B-Y646C**	0.21 ± 0.04 (3) [δ]
**2B-N649S**	2.08 ± 0.48 (4)
**2B-N649T**	0.65 ± 0.14 (4)
**2B-A652G**	1.26 ± 0.30 (5)
**2B-A652P**	0.86 ± 0.21 (4)
**2B-I655F**	0.81 ± 0.22 (6)

The total NMDAR subunit protein expression was expressed relative to WT (mean ± sem, n, number of assay) and was assessed by unpaired Student t-test compared to the same day WT control, [δ] $p < 0.05$. All or part of data from [a] Xu et al. 2021, [b] Lewis et al. 2023, [c] Fernández-Marmiesse et al. 2018, and [d] Amador et al. 2020 are included for comparison.

Supplemental Table S4. Summary of variant NMDAR response time course

	Rise time (ms)	I_{SS}/I_{PEAK}%	FAST tau (ms)	SLOW tau (ms)	% FAST tau	N
**WT GluN1/2A**	8.5 ± 0.5	65 ± 4.0	37 ± 2.2	185 ± 22	82	37
**1-A637S/2A** [δ]	7.0 ± 0.63	77 ± 3.7	44 ± 4.3	152 ± 54	72	7
**1-A637V/2A** [δ]	5.6 ± 0.61	75 ± 7.3	50 ± 5.2	240 ± 47	89	5
**1-G638A/2A** [δ]	8.9 ± 0.54	65 ± 11	67 ± 7.5	423 ± 110	93	5
**1-G638V/2A**	12 ± 3.1	39 ± 3.7	125 ± 8.0	820 ± 222	83	6
**1-M641I/2A** [a]	13 ± 1.8	47 ± 8.6	54 ± 6.3	78 ± 18	44	6
**1-M641L/2A**	15 ± 1.6	28 ± 7.8	68 ± 2.8	310 ± 49	87	6
**1-M641V/2A** [δ]	8.8 ± 0.91	22 ± 8.5	43 ± 5.4	250 ± 56	91	5
**1-I642L/2A** [δ]	4.5 ± 0.20	54 ± 7.0	17 ± 2.3	88 ± 32	69	5
**1-I642T/2A** [δ]	5.4 ± 0.51	59 ± 7.5	21 ± 1.8	121 ± 34	84	5
**1-I643V/2A** [δ]	7.2 ± 0.55	44 ± 11	75 ± 18	1797 ± 1463	93	5
**1-V644M/2A** [δ]	6.1 ± 0.23	73 ± 14	639 ± 146	2132 ± 523	70	5
**1-A645S/2A**	11 ± 1.9	79 ± 6.5	42 ± 4.2	677 ± 61	95	7
**1-N650I/2A** [δ]	5.9 ± 1.3	94 ± 2.7	1078 ± 85	3963 ± 631	69	5
**1-N650K/2A**	10 ± 2.0	76 ± 2.4	183 ± 28	823± 187	84	7
**1-A652T/2A**	9.1 ± 0.7	49 ± 4.8	30 ± 2.2	89 ± 55	52	6
**1-A653G/2A**	15 ± 0.34	64 ± 9.8	298 ± 50	1195 ± 111	76	6
**1-A653T/2A** [δ]	4.4 ± 0.39	67 ± 11	662 ± 159	1769 ± 264	69	5
**1-F654C/2A** [δ]	5.0 ± 1.6	62 ± 9.1	333 ± 72	559 ± 110	63	5
**1-L655Q/2A**	3.5 ± 0.30	50 ± 6.1	172 ± 23	1075 ± 312	73	8
**2A-S632F** [δ]	5.9 ± 0.42	59 ± 7.7	32 ± 6.5	109 ± 43	58	5
**2A-A635T** [δ]	6.6 ± 1.1	57 ± 8.7	158 ± 28	820 ± 170	83	5
**2A-V639I** [δ]	5.8 ± 0.30	84 ± 5.0	444 ± 122	1785 ± 815	70	6
**2A-L642M**	9.5 ± 2.1	64 ± 11	149 ± 16	321 ± 80	63	8
**2A-L642R**	12 ± 0.78	92 ± 1.9	79 ± 9.8	369 ± 91	78	7
**2A-A643D**	37 ± 5.0	84 ± 7.1	669 ± 205	1404 ± 420	47	9
**2A-S644G**	8.5 ± 0.94	66 ± 11	452 ± 126	4721 ± 1823	57	6
**2A-T646A**	21 ± 2.5	98 ± 0.4	1408 ± 165	4764 ± 1176	74	9
**2A-T646R** [δ]	14 ± 1.7	90 ± 3.2	998 ± 133	2058 ± 288	68	6
**2A-N648S**	14 ± 0.88	78 ± 3.0	51 ± 2.8	506 ± 123	85	9
**2A-L649V**	9.0 ± 2.0	95 ± 4.1	2387 ± 1032	3675 ± 887	53	8
**2A-A650S** [δ]	7.8 ± 1.1	63 ± 14	89 ± 16	777 ± 257	87	5
**2A-F652V**	9.2 ± 1.0	67 ± 5.0	127 ± 67	200 ± 51	57	8
**2A-M653I**	13 ± 1.5	75 ± 5.2	42 ± 6.0	503 ± 52	93	7
**2A-M653V**	13 ± 1.1	87 ± 1.8	507 ± 39	1244 ± 146	57	7
**2A-I654T**	14 ± 0.87	91 ± 5.3	613 ± 54	2328 ± 519	77	6
**WT GluN1/2B**	13 ± 1.0	78 ± 2.6	321 ± 34	810 ± 77	67	26
**2B-A639V** [δ]	7.0 ± 1.1	97 ± 1.9	2791 ± 1211	8648 ± 2090	27	5
**2B-I641T** [δ]	7.0 ± 0.52	52 ± 7.8	386 ± 135	1540 ± 197	42	16
**2B-A652G**	12 ± 0.59	78 ± 6.5	385 ± 43	1220 ± 86	82	6
**2B-A652P** [δ]	5.4 ± 0.38	86 ± 0.88	81 ± 9.5	340 ± 64	89	5
**2B-I655F**	15 ± 1.4	91 ± 1.5	180 ± 6.0	993 ± 78	77	9

[δ] Dataset-2 of WT (*see* Supplemental Table S5) was used for the variants as a control. N is the number of cells recorded.
[a] Data from Xu et al., 2021 [43].

Supplemental Table S5. WT controls of NMDAR response time course

	Dataset-1 WT GluN1/2A	Dataset-2 WT GluN1/2A	Dataset-1 WT GluN1/2B	Dataset-2 WT GluN1/2B
**Rise time (ms)**	8.5 ± 0.5	6.5 ± 0.37	13 ± 1.0	11 ± 0.60
**I_{SS}/I_{PEAK}%**	65 ± 4.0	66 ± 5.3	78 ± 2.6	75 ± 4.6
**$Tau_{weighted}$ (ms)**	44 ± 2.2	51 ± 3.6	524 ± 23	663 ± 27
**FAST tau (ms)**	37 ± 2.2	36 ± 4.1	321 ± 34	312 ± 22
**SLOW tau (ms)**	185 ± 22	201 ± 49	810 ± 77	1256 ± 71
**% FAST tau**	82	73	67	60
**N**	37	22	26	20

Two sets of experiments separated by a multi-year interval were used to evaluate the response time course. For each temporally distinct set of experiments, we pooled data from same day controls to obtain WT parameter values for comparison to variants.

Dataset-1 of WT was used as a control for variants GluN1-G638V, GluN1-M641L, GluN1-A645S, GluN1-N650K, GluN1-A652T, GluN1-A653G, GluN1-L655Q, GluN2A-L642M, GluN2A-L642R, GluN2A-A643D, GluN2A-S644G, GluN2A-T646A, GluN2A-N648S, GluN2A-L649V, GluN2A-F652V, GluN2A-M653I, GluN2A-M653V, GluN2A-I654T, GluN2B-A652G, and GluN2B-I655F. Dataset-2 of WT was used as a control for variants GluN1-A637S, GluN1-A637V, GluN1-G638A, GluN1-M641V, GluN1-I642L, GluN1-I642T, GluN1-I643V, GluN1-V644M, GluN1-N650I, GluN1-A653T, GluN1-F654C, GluN2A-S632F, GluN2A-A635T, GluN2A-V639I, GluN2A-T646R, GluN2A-A650S, GluN2B-A639V, GluN2B-I641T, and GluN2B-A652P.

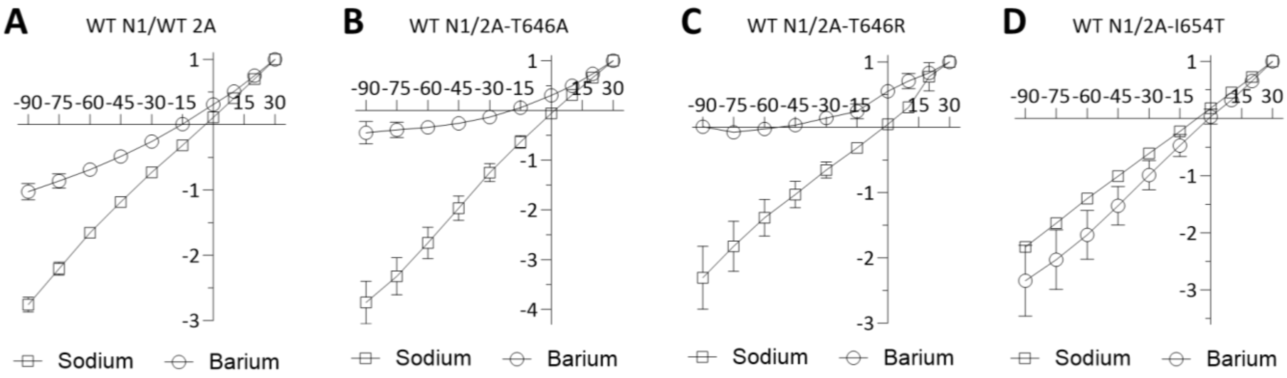

Supplemental Figure S1. M3 variants alter barium permeability. Current-voltage relationships determined in *Xenopus* oocytes are shown for 90 mM NaCl or 60 mM $BaCl_2$ (see Methods) for ***(A)*** WT GluN1/WT GluN2A, ***(B)*** WT GluN1/GluN2A-T646A, ***(C)*** WT GluN1/GluN2A-T646R, ***(D)*** WT GluN1/GluN2A-I654T are shown. *See* Supplemental Table S2 for reversal potentials.

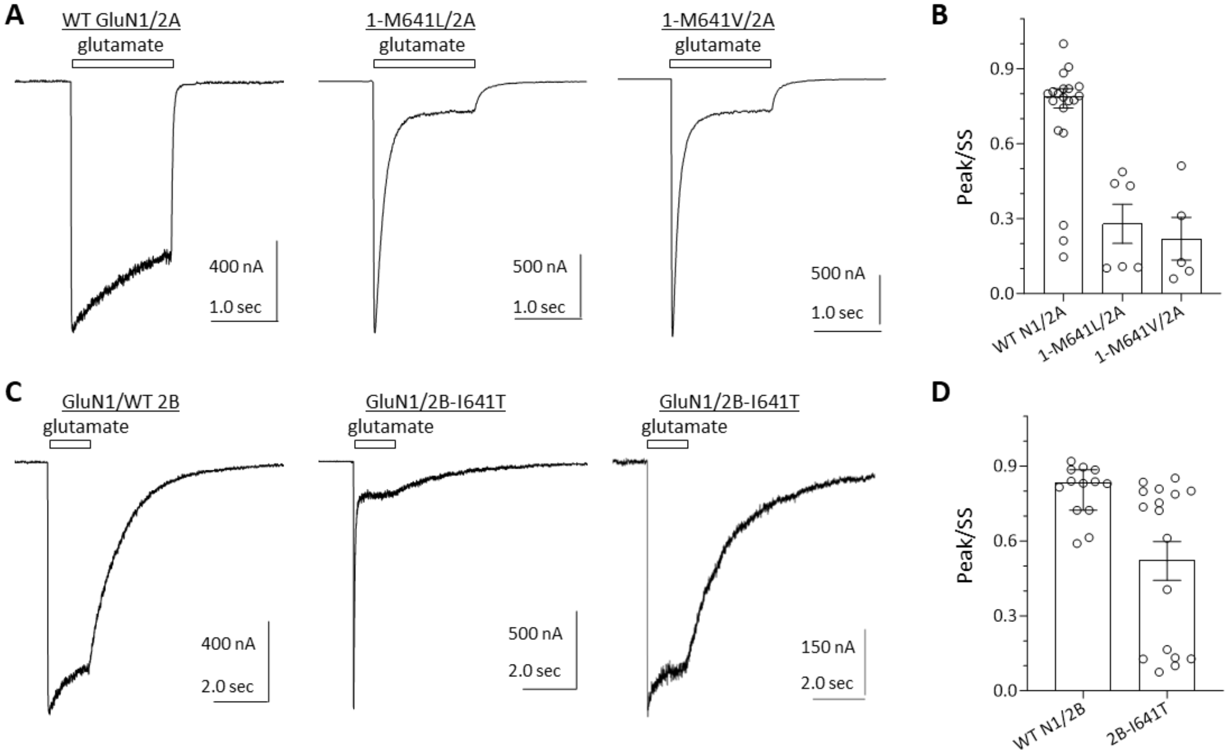

Supplemental Figure S2. M3 variants that alter desensitization. (*A,C*) Representative whole cell current responses recorded under voltage clamp illustrate the deactivation time course for GluN1-M641L/GluN2A, GluN1-M641V/GluN2A, and GluN1/GluN2B-I641T NMDARs in response to 1.5 seconds application of 1 mM glutamate with 100 μM glycine present in all solutions. Two different responses are shown in (***C***) for GluN1/GluN2B-I641T to illustrate the cell-to-cell variability in the degree of desensitization. **(*B,D*)** Summary of the degree of desensitization expressed as the ratio of the amplitude of the peak response to the amplitude of the steady-state response.

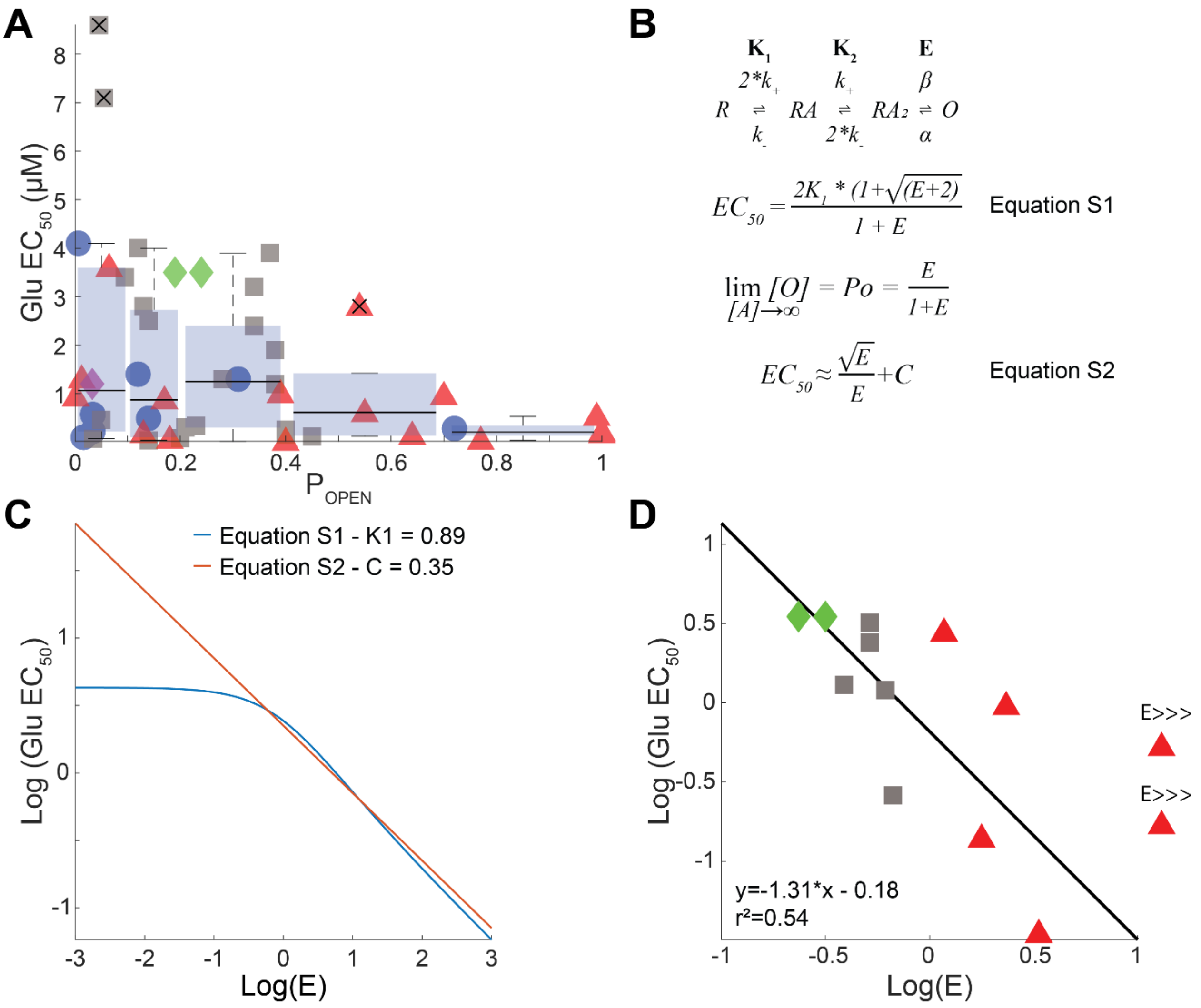

Supplemental Figure S3. Non-linear transformations used to evaluate the correlation between glutamate EC_{50} and maximum open probability (P_O). ***(A)*** Scatter plot and overlaid binned box-and-whiskers plot illustrating the spread of all the variant and WT data demonstrating the potential relationship between P_{OPEN} (x-axis) and glutamate EC_{50} (y-axis). The spread and mean of the glutamate EC_{50} data is high at low Po (0 - 0.4), whereas at higher Po values (0.4 - 1) the spread and mean of the glutamate EC_{50} data is lower. Data points identified as outliers in the binned whisker plots are marked with an *X*. ***(B)*** The theoretical relationship between Po, E (defined as the ratio of the opening rate □ to the closing rate □□, and EC_{50} based on the model is shown (Del Castillo and Katz, 1957; Lester & Jahr, 1992). This simplified model (displayed on the plot) can be solved to determine a direct relationship between E, EC_{50}, and P_O (Equation S1) . ***(C)*** An approximation (Equation S2) of the derived relationship between E and EC_{50} provides a good estimate of this relationship over 4 Log units, which supports the use of the linear regression on the Log-Log transform of the E (converted from the P_O) and EC_{50} of the data. ***(D)*** Linear regression of transformed data (Log E , x-axis; Log EC_{50}, y-axis), results the fit shown in Figure 4F based on the simplified theoretical model. Variants with very high P_{OPEN} values (~1.0) were excluded from the linear regression because their E value approaches infinity (E>>>), which biases the fitting. However, these high P_O remain reasonably fit in the original data space (Po and EC_{50}, *see* Figure 4F).

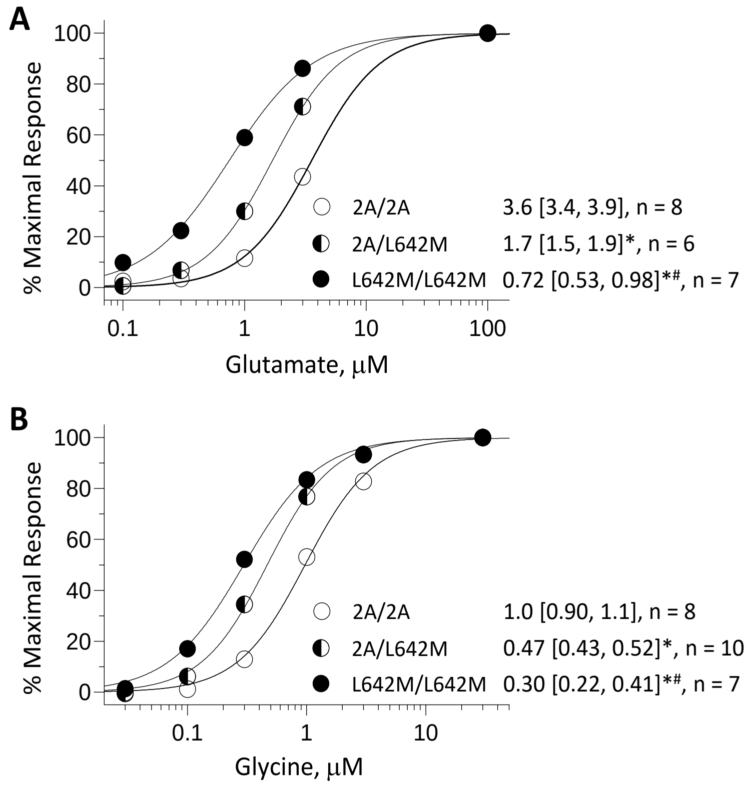

Supplemental Figure S4. Effects of one or two copies of the variant GluN2A-L642M subunit on agonist potency. Composite concentration-response curves for glutamate and glycine at tri-heteromeric receptors GluN1/GluN2A$_{C1}$/GluN2A$_{C2}$ (labelled 2A/2A), GluN1/GluN2A(L642M)$_{C1}$/GluN2A$_{C2}$ (labelled 2A/L642M) and GluN1/GluN2A(L642M)$_{C1}$/GluN2A(L642M)$_{C2}$ (labelled L642M/L642M) expressed in *Xenopus* oocytes. C1 and C2 indicate the coiled coil domains and ER retention signal added to the C-terminus as described by Hansen et al. (2014) and utilized in Yuan et al. (2014). The potency of glutamate (***A***, in the presence of 100 μM glycine) and of glycine (***B***, in the presence of 100 μM glutamate) were evaluated by TEVC recordings on *Xenopus* oocytes at holding potential of -40 mV (*See* Methods). Data was expressed as mean [± 95% CI, confidence interval], number of oocytes. The mean EC_{50} values with 95% CI were determined from the $LogEC_{50}$ values; *indicates 95% CIs that are non-overlapping with WT 2A/2A receptors, and $^{\#}$indicates 95% CIs that are non-overlapping with 2A/2A-L642M receptors, which corresponds to $p < 0.01$.

References

Del Castillo J, Katz B (1957). Interaction at end - plate receptors between different choline derivatives. *Proc Roy Soc Lond B Biol Sci* 146: 369-381.

Hansen KB, Ogden KK, Yuan H, Traynelis SF (2014). Distinct functional and pharmacological properties of Triheteromeric GluN1/GluN2A/GluN2B NMDA receptors. *Neuron* 81(5):1084-1096. doi: 10.1016/j.neuron.2014.01.035. PMID: 24607230

Lester RA, Jahr CE (1992) NMDA channel behavior depends on agonist affinity. *J Neurosci* 12(2):635-43.

Yuan H, Hansen KB, Zhang J, Pierson TM, Markello TC, Fajardo KV, Holloman CM, Golas G, Adams DR, Boerkoel CF, Gahl WA, Traynelis SF (2014). Functional analysis of a de novo GRIN2A missense mutation associated with early-onset epileptic encephalopathy. *Nat Commun* 5:3251. doi: 10.1038/ncomms4251. PMID: 24504326
